# Bridging laboratory and field research: Method adjustments to blood feed field-derived *Aedes aegypti*

**DOI:** 10.1371/journal.pntd.0013339

**Published:** 2026-05-18

**Authors:** Yanouk Epelboin, Leonardo Daniel Ortega-Lopez, Emilie Balthazar, Alaïs Cornement, Amandine Guidez, Isabelle Dusfour, Mathilde Gendrin

**Affiliations:** 1 Microbiota of Insect Vectors Group, Institut Pasteur de la Guyane, Cayenne, French Guiana; 2 UA17, Santé des Populations en Amazonie, lnserm, Cayenne, French Guiana; 3 Unité d’Entomologie Médicale, Institut Pasteur de la Guyane, Cayenne, French Guiana; University of California Davis, UNITED STATES OF AMERICA

## Abstract

Reference strains of *Aedes aegypti*, reared over decades under laboratory conditions, are commonly used in research due to their consistency and ease of handling. While their use is relevant in terms of reproducibility between labs, complementary work on field-collected mosquitoes and their progeny is essential to capture biological and behavioral variations of natural populations. However, experimental set-ups optimized on reference strains are not always successful with field-derived mosquitoes; their lack of attraction to experimental blood meals is a recurrent issue. In this study, we evaluated methods to improve blood-feeding rate of field-derived mosquitoes from Cayenne (French Guiana), using the New Orleans reference strain as a control. We tested the impact of several blood-feeding systems for mosquitoes kept in a cage or in individual vials and adjusted starvation parameters. Individual mosquitoes offered a blood meal in lids of microtubes had the highest blood-feeding rate. For field-derived mosquitoes, starvation, or alternatively, provision of a 3% salt solution instead of the sugar solution, also consistently improved blood-feeding rate, with a minimal impact on survival. Our results may be helpful for establishment of new colonies, including in resource-limited settings, studies on fitness of field-derived mosquitoes and on experiments requiring individual level monitoring.

## Author summary

*Aedes aegypti* is the major vector of dengue virus, causing hundreds of millions of cases per year. To fight against disease transmission, mosquitoes are studied in the laboratory, notably using colonies of mosquitoes kept over decades in the laboratory. These reference strains have been selected to fit laboratory conditions and may thus not fully reflect the traits of natural populations. While working on such strains is relevant in a sake of consistency between labs and ease of handling, it is also important to work on field-collected mosquitoes and their progeny to capture more representative biological and behavioral variation. However, experimental set-ups that have been optimized on reference strains are not always successful with field-derived mosquitoes; their lack of attraction to experimental blood meals is a recurrent issue. In this study, we adjusted methods to improve blood-feeding rate of field-derived mosquitoes from Cayenne (French Guiana), using the New Orleans reference strain as a control. We tested the impact of several blood-feeding systems for mosquitoes kept in a cage or in individual vials and adjusted starvation conditions. Our data indicate that the reference strain blood feeds relatively well and is not much impacted by experimental adjustments, while blood feeding of field-derived mosquitoes is substantially affected by blood container and starvation. Our results may enhance experimental reliability of colony establishment and maintenance, including in resource-limited settings and of studies on the fitness of field-derived mosquitoes, including at the individual level.

## Introduction

*Aedes aegypti* is a major vector of arboviruses that strongly impact global health. This mosquito species is implicated in transmission of dengue, yellow fever and the recently emerged chikungunya and Zika viruses. Among them, dengue outbreaks occur annually in tropical regions causing an approximate burden of 390 million cases per year [1,2]. Between 2010 and 2019, chikungunya and Zika viruses increased in prevalence and caused outbreaks of more than 100,000 clinical cases worldwide [3]. While yellow fever has been less prevalent in recent years, it caused an estimate of 109,000 cases in Africa and South America in 2018, half of them leading to death [4]. While vaccines against these arboviruses are under development [5] or exist (yellow fever) [6], vector control is still essential to prevent their transmission [7].

Studying the ecology and biology of *Ae. aegypti* is essential to understand the key factors that contribute to its success as a vector and thus to enhance development of targeted and effective vector control strategies preventing arboviral transmission. Notably, environmental factors including temperature, humidity, light exposure and nutrient availability may influence key ecological and biological traits such as mating dynamics, population growth, and fecundity [8]. Determining causal effects of such environmental factors on life history traits is however extremely complex with wild populations in field conditions due to natural variability in environmental variables and between individuals – linked to diet, age, genetics, and body size [9]. Therefore, conducting experiments in controlled laboratory conditions is useful to establish the relative importance of environmental variables on life history traits.

Reference laboratory strains of *Ae. aegypti* have enabled determination of standard phenotypes and genotypes, which is essential to understand their biology and their interactions with pathogens. However, despite the advantages of maintaining laboratory mosquito colonies to determine causal influences of the environment on various traits of *Ae. aegypti*, findings are not always transposable to wild-type populations [10]. This discrepancy might be due to long-term adaptations of laboratory colonies that affect their survival and reproductive fitness [11]. Inbreeding, bottle-necks and genetic drift are some of the underlaying causes of such differences [11,12]. Experimental designs must address this issue to ensure findings are relevant and applicable to natural populations, notably for studies focusing on the influence of genetic variability on life history traits or mosquito microbiota.

While most of the earlier studies on mosquito blood feeding were based on the use of live animal hosts, artificial feeding systems are increasingly used to follow the principles of Replacement, Reduction, and Refinement (the 3Rs). They aim to find alternatives to animal testing, to optimize the amount of information obtained from fewer animals, and to adopt methods that alleviate distress respectively [13]. Various artificial feeding techniques were gradually developed including membrane and capillary feeding, based on natural or engineered biocompatible materials [14–16]. Membrane feeding is commonly used when mass-rearing *Ae. aegypti* [17], generally with glass membrane feeders [18] or Hemotek systems (Discovery Workshops, Accrington, UK) which maintain blood temperature [19]. In recent years, several devices made from common and low-cost lab materials have been used to feed mosquitoes [20–22] and appear to be adapted for laboratory mosquitoes [23]. Various blood sources (human, avian, cattle, etc.), and membrane types (chicken skin, collagen, latex gloves and Parafilm) have been tested to optimize blood feeding [14,16]. Sugar starvation has been described as a key factor to increase blood-feeding rate (proportion of blood fed individuals), the results vary depending on the species, strain, and, most notably, the duration of the sugar starvation period [24]. Protocols are however not always transferable from reference laboratory strains to field-collected mosquitoes, so method adjustments are required to improve blood feeding of the latter.

In this context, this study aimed to investigate the physiological and behavioral differences between the New Orleans laboratory strain and the first laboratory progeny (F1) from a population of *Ae. aegypti* mosquitoes collected in Cayenne, through two key objectives. First, we compared the efficacy of different blood-feeding methods with various types of blood containers. Second, we evaluated the effects of time and type of starvation on blood-feeding success. Together, these investigations improve the current understanding of the factors influencing experimental outcomes in *Ae. aegypti* derived from the field under controlled conditions. Our low-tech methods can be used in conventional laboratory conditions as well as in field-based laboratories.

## Results

### Comparison of blood-feeding techniques

**Fig 1:**
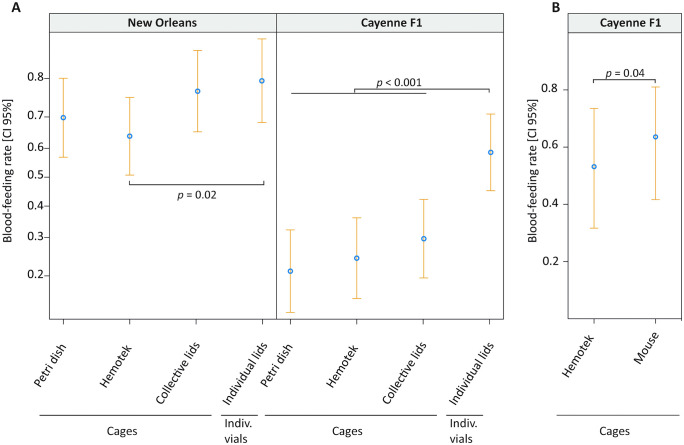
Effect of blood-feeding technique on the blood-feeding rate of New Orleans strain vs field-derived Ae. aegypti mosquitoes (Cayenne F1). **A.** Effect of blood-feeding methods on the proportion of blood-fed females after 30 min depending on the blood container. All feeding methods deliver human RBCs mixed with human serum; more technical details are provided in the Methods section of the main text (n = 47-51 females for each condition; experiment was performed in triplicate). **B.** Comparison of blood-feeding rates in Cayenne F1 mosquitoes between artificial Hemotek feeding-system containing bovine blood and anesthetized mice (n = 85 to 100 females per cage received a blood meal. This experiment was conducted in 7 cages per feeding condition on two separate dates). Blue dots represent mean predicted values from the final models and yellow bars represent 95% confidence intervals. More details are presented in S1 File.

We first quantified the difference in blood-feeding rate between *Ae. aegypti* mosquitoes of the New Orleans reference strain and the progeny of *Ae. aegypti* mosquitoes collected as larvae, referred to here as “Cayenne F1”. We compared several blood-feeding methods: we offered blood in a Hemotek capsule, in a Petri dish, in several lids of microtubes laid on the cage (‘Collective lids’) or in one lid offered to a mosquito kept in an individual tube (‘Individual lids’, Table 1). These containers were placed upside down on the cages so that mosquitoes could bite through an artificial membrane (Table 1). The analysis revealed a significant effect of the interaction between mosquito strains and the blood-feeding technique on the blood-feeding success (*X*^*2*^_(3)_=11.26, *p* = 0.01, Fig 1A and Table A in S1 File), which indicates that the efficiency of blood-feeding methods differed depending on mosquito origin. Specifically, this model predicted a mean feeding rate of 0.73 (95% CI: 0.63-0.81) for the New Orleans strain, and of 0.32 (95% CI: 0.23-0.43) for Cayenne F1. When we offered for instance blood in Petri dish, we observed a strong effect of the mosquito strain, with a mean feeding rate of 0.7 for New Orleans and only 0.21 for Cayenne F1 (Fig 1A; *β* = -2.07, Z = -5.11, *p* < 0.01; Table A in S1 File for fitted values). Regarding the blood-feeding method, most Post-Hoc pairwise comparisons did not differ significantly from one another within the New Orleans strain (Fig 1 and Table B in S1 File). However, Post-Hoc pairwise comparisons of blood-feeding methods within the Cayenne F1 strain showed that individual lids resulted in the highest blood-feeding rate (0.59, 95% CI: 0.45-0.71; Fig 1A; *p* < 0.001; Table B in S1 File), compared to the other methods such as collective lids (0.30, 95% CI: 0.20-0.42), Hemotek (0.24, 95% CI: 0.15-0.36), and Petri dish (0.21, 95% CI: 0.13-0.32).

**Table 1:** Characteristics of each of the blood-feeding systems used in the experiments. The condition shown in bold was used for the starvation experiment.

Treatment	Type of capsule	Type of membrane	Volume of blood	No. of blood aliquots/ expt	Required volume of blood/ expt
Collective 1	Hemotek	Collagen membrane	3 mL	1	3 mL
**Collective 2**	**Petri dish 35x10 mm**	**Parafilm membrane**	**4 mL**	**1**	**4 mL**
Collective 3	15 lids of 1,5 mL microtube	Parafilm membrane	225 µL	15	3.4 mL
Individual 1	1 lid of 1,5 mL microtube per mosquito	Parafilm membrane	225µL	50	11.3 mL

In another experiment, we also compared anaesthetized mice and bovine blood in Hemotek as blood-feeding sources. Females of Cayenne F1 *Ae. aegypti* reached a predicted blood-feeding mean rate of 0.64 (95% CI: 0.42-0.81) on mice, slightly higher than their rate of 0.53 (95% CI: 0.32-0.73) on Hemotek devices (*X*^*2*^_(1)_=4.33, *p* = 0.04, Fig 1B).

### Effect of starvation on blood-feeding rates

**Fig 2:**
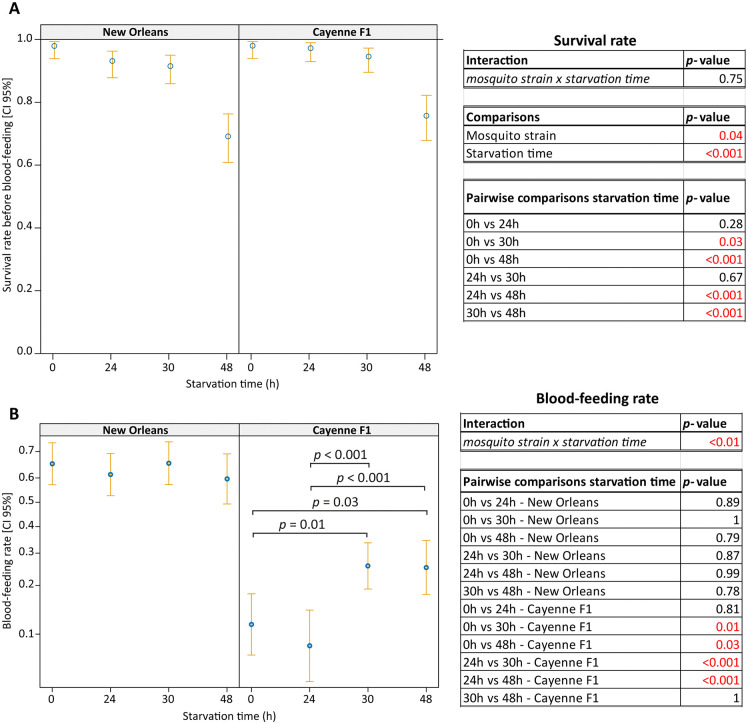
Adjustment of starvation time prior to blood feeding in New Orleans and Cayenne F1 Ae. aegypti. In this figure, starvation corresponds to total deprivation (no water, no sugar). **A.** Effect of starvation time on mosquito survival at the time of blood feeding. **B.** Effect of starvation time on mosquito blood-feeding rate. In A-B, blue dots represent the mean predicted values from final models and yellow bars represent 95% confidence intervals; n = 40-50 females per condition per replicate; 3 replicates. Statistical analyses are presented in the associated tables. More details are presented in S1 File.

We starved mosquitoes 0 to 48h for sugar and water (total deprivation) before offering them blood in Petri dishes. When analyzing mosquito survival, we found that there was no significance from the interaction term between *Ae. aegypti* origin (Cayenne F1 and New Orleans) and any of the four pre-blood-feeding starvation times (*X*^*2*^_(3)_=1.21, *p* = 0.75). However, we found significant effects from both independent variables separately. Such was the effect of the *Ae. aegypti* strain (*X*^*2*^_(1)_=4.19, *p* = 0.04; Fig 2A), and of the pre-blood-feeding starvation time (*X*^*2*^_(3)_=111.71, *p* < 0.001; Fig 2A) on survival rate. Regarding *Ae. aegypti* strains, New Orleans strain survival had a mean predicted value of 0.92 (95% CI: 0.89-0.94) and Cayenne F1 of 0.95 (95% CI: 0.92-0.96). Post-hoc analyses showed that 48h of starvation significantly reduced *Ae. aegypti* survival (*β* = -2.93, Z = -6.74, *p* < 0.001); Table C in S1 File).

Considering blood-feeding rate, both mosquito strains behaved differently according to starvation time (interaction between *Ae. aegypti* strain and starvation time: *X*^*2*^_(3)_=14.81, *p* < 0.01, Fig 2B). From Post Hoc analyses, we observed no significant effect between any of the pairwise comparisons of the blood-feeding methods for New Orleans mosquitoes (Post Hoc pair-wise comparisons: Table D in S1 File). For Cayenne F1 however, the mean predicted blood-feeding rate increased from 0.12 at 0h of starvation (no starvation, 95% CI: 0.07-0.18) to 0.26 at 30h (95% CI: 0.19-0.34; estimate = -0.98 ± 0.32 SE, Z = -3.02, *p* = 0.01) and to 0.25 at 48h (95% CI: 0.18-0.35; estimate = -0.95 ± 0.34 SE, Z = -2.76, *p* = 0.03). Blood-feeding rates at 24h (0.08, 95% CI: 0.05-0.14) were also significantly lower than at 30h (estimate = -1.33 ± 0.36 SE, Z = -3.71, *p* < 0.01) and 48h of starvation (estimate = -1.3 ± 0.38 SE, Z = -3.46, *p* < 0.01; Table D in S1 File).

Taken together, these data suggested that starvation was ineffective to increase blood-feeding rates in New Orleans *Ae. aegypti* strain, while a 30h-starvation was beneficial to increase blood-feeding rates in Cayenne F1 *Ae. aegypti* strain, having little effect on their mortality.

We further tested whether hunger or thirst was the essential driver to blood feeding, providing mosquitoes with water only or with a hyperosmotic aqueous solution containing 3% NaCl, keeping 10% sugar solution and total deprivation as non-starved and fully starved controls. In this experiment, starved and non-starved mosquitoes were proposed a blood meal at the same time to account for potential differences due to circadian-rhythm related issues. We selected relevant conditions from the previous experiment, focusing on Cayenne F1 mosquitoes and on 24h and 30h timings.

**Fig 3:**
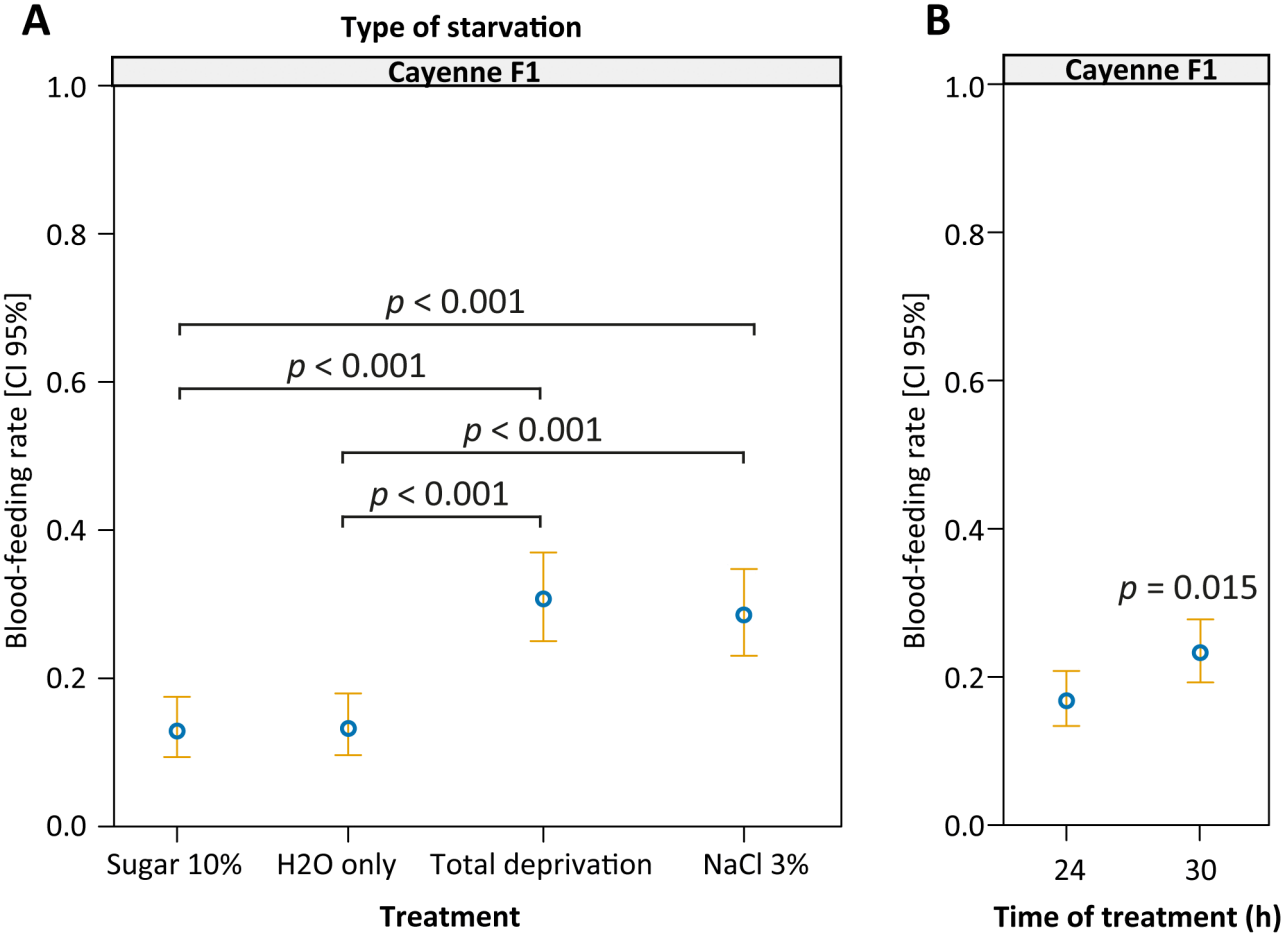
Effect of type and time of starvation on blood-feeding success. **A**: Effect of the type of treatment before blood feeding. Mosquitoes were exposed for 24h or 30h to sugar 10%, water only, NaCl 3% or were totally deprived. **B**: Effect of starvation time on blood-feeding success. In A,B, blue dots represent the mean predicted values from final models and yellow bars represent 95% confidence intervals; n = 48-55 females per condition per replicate; 3 replicates. More details of statistical analyses are presented in S1 File.

We found that starvation time had a significant impact on mortality (*X*^*2*^_(1)_=4.21, *p* = 0.040), but that mortality remained low at 30h (0.062 ± 0.014). Starvation type and time both significantly affected blood-feeding rates (starvation type: *X*^*2*^_(3)_=49.65, *p* < 0.001; time: *X*^*2*^_(1)_=5.90, p = 0.015; Fig 3). As there was no significant interaction between starvation type and time, we performed post-hoc analyses on starvation types, pooling mosquitoes from both time points. We found that mosquitoes provided with water only did not feed better than non-starved mosquitoes (*p* = 1.0), while total deprivation and feeding with salt solution significantly affected blood-feeding rate (*p* < 0.001) to a similar extent (total deprivation vs NaCl solution: *p* = 0.94; Table E in S1 File). This indicates that thirst rather than lack of energy drives attraction to blood feeding, at least until 30h.

We observed a slight discrepancy between Figs 2 and 3 on the impact of total deprivation at 24h. Compiling together our data from Cayenne F1 that were not starved or totally deprived during 24h or 30h, we found that a 30h starvation highly significantly affected blood-feeding rate (*X*^*2*^_(2)_=38,89, *p* < 0.0001; Table F in S1 File) while a 24h starvation had a marginally significant impact (*p* = 0.088; S1 Fig; data from Figs 2 and 3). The difference between a 24h and a 30h starvation was strongly significant (*p* = 0.0006). In these compiled data, starvation time again induced a significant yet minor mortality (24h: 0.011 95%CI [0.0037- 0.034]; 30h: 0.046, 95% CI [0.023-0.91]). To account for potential effects of circadian rhythms, we tested whether non-starved mosquitoes fed in the morning (9 am for 24h) and afternoon (3 pm for 30h) had a different blood-feeding rate (data from Fig 3). This schedule did not significantly affect feeding rate (*X*^*2*^_(1)_=1.79, *p* = 0.18), suggesting that the difference in feeding rate between 24h and 30h starvation was largely linked to prolongation of starvation itself.

## Discussion

Blood-feeding success of mosquitoes is a fundamental aspect of their fitness; its optimization may notably be intended to increase the yield of rearing or to improve the representativeness of the sample in experiments requiring blood feeding, such as vector competence studies. Our findings reveal differences between the New Orleans laboratory strain and the first progeny of field-collected *Ae. aegypti* mosquitoes that have important implications for experimental design.

While adjusting protocols to increase blood-feeding rates in Cayenne F1 *Ae. aegypti*, we confirmed that the F1 of our local population can feed on artificial blood-feeding devices. Feeding on mice was slightly more efficient than on Hemotek, yet the difference was too minor to justify the use of animals, considering ethical reasons and logistical drawbacks. As described in other studies, simple systems can be used with affordable materials, *e.g.,* cardboard cups, Parafilm membrane, and simple heating systems as alternatives to the commercially available Hemotek system [20,22,25,26]. We monitored a similar blood-feeding rate with the use of Petri dish covered with a hand warmer as with Hemotek capsules. Such self-made systems can be particularly useful for several applications, including routine experiments in laboratories, resource-limited settings, or field experiments. Lids of microtubes were even more efficient than larger containers to support mosquito feeding, especially for individualized mosquitoes. This may be due to mosquito confinement reducing distance to blood source. We observed a higher success with 15 microtube lids than with a Petri dish while both had a blood surface area of approximately 11 cm^2^. We hypothesize that the dispersion of the 15-lid blood on the cage grid explains this greater success, as it overall covered a greater area. Feeding on microtube lids offers an effective and low-cost approach for experiments requiring precise mosquito development or individual life history traits studies. A key advantage of this system is also its capacity to deliver blood meals to mosquitoes in a sterile environment, which is essential for experimental designs involving microbiota manipulation [27]. Additionally, microtube lids limit blood usage compared to other methods, making them particularly useful for resource-limited contexts. Preparing microtube lids in large numbers is however labored and time-consuming, hence we prioritized the use of Petri dishes when evaluating methods, for its convenience and availability.

Comparing different types of starvation, we showed that thirst rather than lack of energy was the main driver for mosquito attraction. Indeed, providing water only completely abrogated the impact of starvation. On the contrary, mosquitoes provided a hyperosmotic NaCl solution, with a salt concentration close to sea water, were as attracted to blood as completely starved mosquitoes. The strong differences observed between types of starvation highlight the importance of specifying how starvation is conducted in scientific manuscripts. For instance, indications such as “sugar starvation” or “sucrose deprivation” require clarification. The results observed with salt water are intriguing; we are not sure that mosquitoes indeed ingested the 3% salt solution and became thirsty via a “crisps and peanut effect” or if they were just water deprived. Yet, we observed mosquitoes on the cotton delivering the salt solution. The presence of salt in water (1%) has been reported not to affect mosquito survival, whether in the presence or absence of sugar [28]. This suggests that salt water, or potentially water containing salt and sugar, may allow to starve mosquitoes while avoiding death by desiccation. Further work would be required to validate this and to check that, for each experimental set-up, salt does not act as a confounding factor.

We found that a 30h starvation significantly increased blood-feeding rate of Cayenne F1 from 0.11 to 0.30 (S1 Fig), with a minimal effect on mosquito survival (0.045). Previous studies have shown that sugar starving of 6 to 24h improves blood-feeding rates in *Aedes* or *Anopheles* [25,29–31]. Conversely, other traits such as host-seeking behavior, appear unaffected by sugar deprivation in female *Ae. aegypti* mosquitoes [24]. Our data suggest that the difference observed after 24h and 30h of starvation is not significantly linked to circadian rhythms of the mosquito according to the feeding rate of non-starved mosquitoes. While *Ae. aegypti* is overall diurnal [32,33], its activity patterns in the field are bimodal, with peak activity occurring at 7:00–8:00 a.m. and 7:00–8:00 p.m. [34–37]. Recent studies on laboratory-reared mosquitoes suggested a relatively strong feeding throughout the day, with a drop during ~3 h in the middle of the day that is driven by the mosquito internal clock [38,39]. Both times tested (9:00–9:30 a.m. and 3:00–3:30 p.m.) fall outside these highs and lows in activity, which may explain why we did not detect any time-related difference.

For New Orleans mosquitoes, our data indicates that starvation is unnecessary and that feeding techniques have a minimal impact. This indicates that New Orleans strain has undergone adaptation to laboratory conditions, enabling more efficient feeding through artificial membranes without requiring additional stimuli such as starvation. This may not be surprising as laboratory environments exert different selective pressure compared to field environments, as laboratory diet is abundant, temperature and humidity are relatively controlled [10], yet artificial feeding lacks natural stimuli and the microbiota may be unbalanced [40,41]. While this effect is detectable with *Ae aegypti*, some traits have been surprisingly well conserved over the years, such as circadian peaks of activity [38,39] and resistance to starvation (as shown here). Considering the anthropophilic behavior and its tendency to breed in artificial containers, we can assume that other mosquito species may undergo even more stringent selection during colonization.

The use of adenosine triphosphate (ATP) has been shown to encourage blood feeding in *Ae. aegypti* [42,43]. ATP is too unstable in aqueous solutions for storage at room temperature, and it is relatively expensive, so we did not use this phagostimulant, except for the comparison between Hemotek and live mouse. However, future studies could investigate its effects in combination with other attractants like lactic acid or aldehydes in human sebum. For instance, ketoglutaric acid, or lactic acid are present in animal odors and may be responsible for mosquito attraction [44]. Human sebum composition and long-chain aldehydes such as decanal and undecanal may also be responsible for mosquito preference in host seeking [45]. We did not assess the impact of blood-feeding conditions on fecundity and fertility parameters, as studies using similar homemade systems observed no significant differences in egg-laying or hatching rate [16,20,22,26].

Vector biology experiments range from basic research on reference strains to applications such as the release of mosquitoes or the implementation of vector control strategies. Between these extremes, a whole set of intermediate set-ups exists and are being developed to ensure a step-by-step path from discovery to application. This includes the use of field-derived mosquitoes, variable microbial set-ups, diverse types of cage sizes or other aspects of mosquito husbandry [40,46]. In this context, our study provides valuable methodological adjustments to work with field-derived *Ae. aegypti*. We used Cayenne F1 as a model, which derives from several collections (one per replicate) in the town of Cayenne. Local adaptation and genetic variability between distinct field populations can however influence physiological and behavioral traits [40], so the observed differences between New Orleans and Cayenne F1 may not be fully generalizable to other field populations. Overall, our results may either be used as such, using the parameters that we found most relevant to study field-derived mosquitoes, or as a framework for method improvement, as they show some important factors affecting the quality of fitness studies.

Beyond the adjustments proposed here, other experimental set-ups can improve work with field mosquitoes. It is preferable to increase the number of mosquitoes collected in the field and to provide them ideal conditions, notably recurrent blood feedings with several blood sources to obtain a large progeny [47]. These adjustments may be useful for colonization and for studies on vector competence or insecticide resistance, which require a large number of mosquitoes. A key advantage of this system is also its capacity to deliver blood meals to mosquitoes in a sterile environment, which is essential for experimental designs involving microbiota manipulation.

In conclusion, our study underscores the need to adapt experimental conditions when working with field mosquitoes. Our data indicates that a 30h starvation, whether of full deprivation or of feeding with salt water, allows best feeding rates. Individualization appears to positively impact feeding, potentially due to a low distance between mosquito and blood. These insights may improve more efficient colony maintenance and experimental reliability, especially in low-resource or field-based laboratories.

## Methods

### Ethics statement

For Fig 1B and some blood meals of New Orleans colony maintenance, anaesthetized mice have been used for mosquito blood feeding. Protocols have been validated by the French Direction générale de la recherche et de l’innovation, ethical board # 089, under the agreement # 973021.

### Mosquito strains

Our reference strain of *Ae. aegypti*, New Orleans, is a well-established laboratory colony routinely maintained in our facility for more than 10 years. Cayenne F1 mosquitoes are the first progeny of mosquitoes collected as larvae and pupae (considered as the parental generation) from 3 to 5 different artificial breeding sites in Cayenne (French Guiana, South America). Larvae were subsequently reared in the laboratory until adult stage, blood fed, mated and were allowed to lay eggs. Multiple larvae collections were carried out to obtain the mosquitoes necessary for the experiments presented here. Both types of mosquitoes are referred to as “strains” throughout this text in a sake of convenience but Cayenne F1 do represent variable local genetic backgrounds.

### Mosquito rearing

Field-collected larvae and pupae from Cayenne were kept in their breeding water and larvae were fed on brewer’s yeast tablets (Gayelord Hauser) until pupal stage. Pupae were removed from breeding trays daily and transferred to plastic containers with tap water in rearing insect cages (Bugdorm, 30 x 30 x 30 cm) to allow adult emergence. Adult male and female mosquitoes were offered a 10% (w/v) sucrose solution through a soaked cotton which was replaced every 3 days. To obtain eggs, female mosquitoes were blood fed twice a week during 30 min on an artificial membrane feeding system Hemotek (6W1, Hemotek Ltd, UK) with heparinized bovine blood provided by the slaughterhouse of Rémire-Montjoly (French Guiana). A plastic container half-filled with dechlorinated tap water and covered on the inner surface with a semi-immerged filter paper was placed inside rearing cages to allow egg-laying. Filter papers were left for 4 days after each blood meal and subsequently removed to dry out in the insectary. Filter papers with eggs were kept dry in zip lock bags until use 1 week to 2 months later. Rearing conditions included a daily temperature of 28 ± 3°C, a relative humidity of 80 ± 10% and a natural 12:12 h light:dark cycle.

In order to standardize the age of mosquitoes for each experiment and synchronize hatching, filter papers containing eggs were submerged into about 200 mL of tap water and subsequently hatched inside a vacuum desiccator for 30 min. Eggs of Cayenne F1 and New Orleans were hatched separately at the same time. Larvae were then placed in rearing trays and were fed with brewer’s yeast tablets (Gayelord Hauser) until pupal stage. Thus, pupae were collected and transferred into cages for adult emergence. For experiments assessing blood-feeding methods efficiency, adult mosquitoes were sugar fed ad libitum on cotton soaked in 10% sucrose solution from the first day of emergence until 24 h before the start of the experiment, or at specific timings for starvation experiments.

### Blood mix preparation

Blood used for experiments corresponds to a 1:1 (v:v) mix of human red blood cells provided by Etablissement Français du Sang (Pointe-à-Pitre, Guadeloupe) with human serum provided by Etablissement Français du Sang (Marseilles, France). Packed red blood cells were washed 3 times with one volume of phosphate buffer saline solution. Supernatant was discarded after a 3900-g centrifugation for 3 min.

### Blood-feeding method comparison

**Fig 4:**
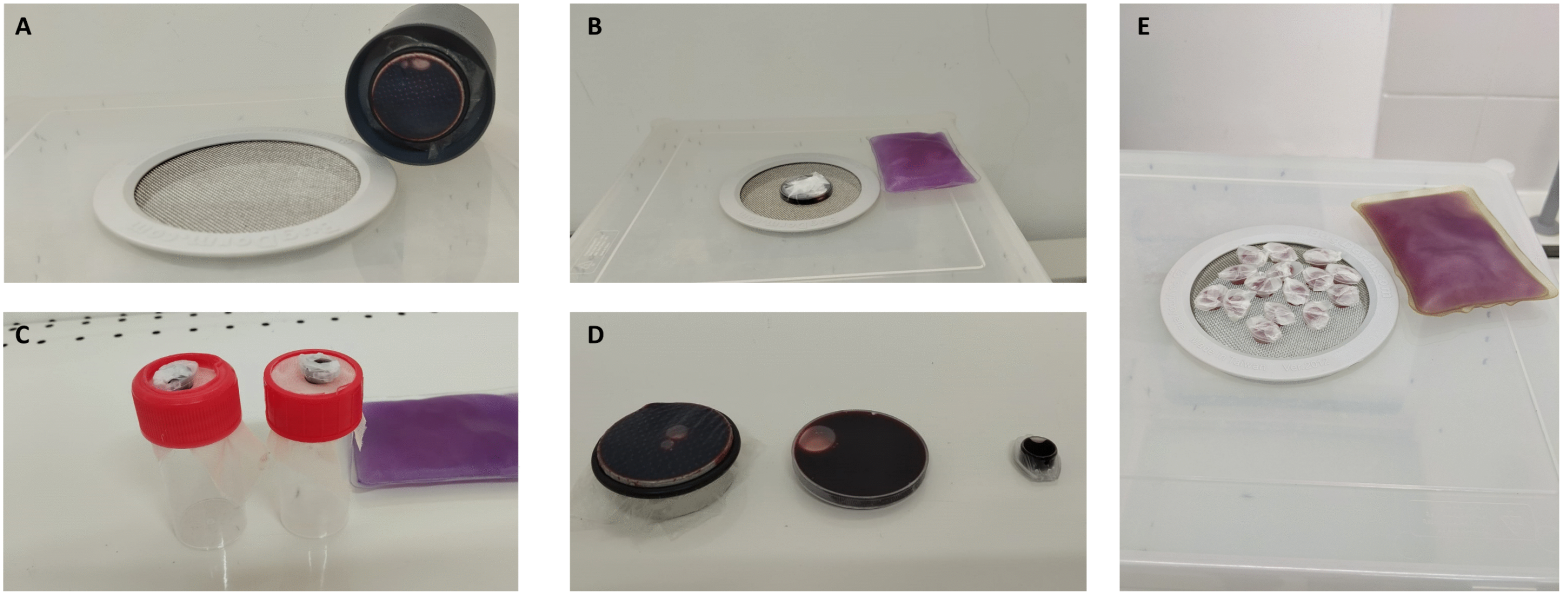
Pictures of the different blood-feeding methods. A: Hemotek system. B: Petri dish of 35x10 mm with a hand warmer. C: Isolated mosquito feeding on an individual 1.5 mL microtube lid with a hand warmer. D: Overview of the three types of blood capsules: Hemotek, Petri dish and lid of 1.5 mL microtube. E: Set of fifteen lids of 1.5 mL microtube with a hand warmer. During blood feeding, the Hemotek membrane lays on the grid in A, and hand warmers lay on top of the blood containers in B-D to maintain temperature.

Three treatments of group blood feeding and one treatment of individual blood feeding were performed for each of the two mosquito strains (Cayenne F1 and New Orleans). Each experimental trial was performed in triplicate. Three groups of about 50 five-day-old females were collected from one rearing cage of each mosquito strain and placed into three adult rearing cages for group blood feeding. We used three different blood-feeding systems for batch feeding (Fig 4): Hemotek, a Petri dish, and a set of 15 microtube lids (treatment called “collective lids” – meaning collective blood feeding). These methods were selected for several reasons. Firstly, the Hemotek system, which is a standard method for feeding mosquitoes used in many insectaries worldwide, allows for precise control of blood temperature and is typically used with a collagen membrane. However, it is limited when it comes to conducting multiple feedings simultaneously or working in laboratories with scarce equipment. In comparison, we chose Petri dishes, as they are accessible and inexpensive in laboratory settings. Microtube lids, very common laboratory consumables, were used to test methods for monitoring individual mosquitoes. Petri dishes and microtube lids were covered with Parafilm, stretched along two perpendicular axes when setting up. Using several caps on large cages allowed for a comparison with the two other methods on cages described above. Following the volumes described in Table 1, blood was distributed among the feeder devices, which were then covered with the corresponding membranes and placed facing downwards on top of the rearing cages. Female mosquitoes could access it by biting through the membrane.

Meanwhile, 50 additional females were also collected from each rearing cage and kept individually for individual blood feeding. They were allocated into 25 mL plastic vials whose inner surface had previously been scratched with sandpaper to allow mosquitoes to grip, covered with a mosquito net on top. Single microtube lids were filled with 225µL blood, covered with Parafilm membrane and then placed downwards on top of each vial for individual mosquito blood feeding (treatment called “individual lids”- meaning individual blood feeding).

Except for Hemotek that had its own electronic heating system at 37°C, all other treatments were kept warm with gel hand warmers of sodium acetate that were left on top of the blood-feeding devices after getting activated. A paper towel was placed between Petri dishes or lids and hand warmers. The hand warmers activate within seconds, reaching 39 ± 1°C and maintaining this temperature throughout the 30 min blood meal. Unlike the metal capsule of the Hemotek system that allows the blood to reach a temperature of 37°C within 3 min, when using a plastic Petri dish or a tube lid, the blood reaches a temperature of 36°C within about 10 min and 3 min respectively.

Before blood meal, dead females were removed from the cages in Fig 1. All mosquitoes were offered blood through their corresponding feeding devices for 30 min to allow enough time for blood feeding (Fig 1). At the end of the experimental time, all fully engorged females from each treatment were counted. Experiment was performed in triplicate.

For blood feeding on mice vs Hemotek, *balb/c* mice (*Mus musculus*) were anaesthetized with a ketamine/xylasine mixture and offered to *Ae. aegypti* females (Cayenne strain) for blood feeding. In parallel, the blood-feeding with Hemotek system was performed in the same condition as described above but instead of human serum, blood was mixed with fetal bovine serum (Sigma-Aldrich) and ATP 3.3% (Sigma-Aldrich). For these experiments, all female mosquitoes were from 7 to 9 days old post emergence. Experiments were conducted on two separate dates, and all mosquitoes were divided into 14 separate cages, with 7 cages for each blood condition. Each cage contained between 80 and 100 females initially and overall, 462 and 493 female *Ae. aegypti* were offered mice and blood in Hemotek respectively. *Ae. aegypti* females were allowed to blood feed for 30 min.

### Impact of total deprivation on blood-feeding rates

Four durations of starvation before blood feeding were tested: 0h (no sugar starvation), 24h, 30h, and 48h. The different deprivation periods were selected based on the literature (typically 24h) and to accommodate feeding the mosquitoes at convenient timings in the morning and the afternoon. For each treatment, 50 five-day-old adult females from each *Ae. aegypti* strain were selected and placed in separate cages. Depending on the starvation duration, female mosquitoes were either immediately offered a blood meal for 30 min or starved for the specified period before being offered the blood meal for the same duration. All females were offered a blood meal using a 35mm-wide and 10 mm-deep Petri dish covered with Parafilm with the corresponding technical details as given in Table 1. Survival was monitored at the start of blood feeding. Experiment was performed in triplicate.

### Impact of deprivation treatments on blood-feeding rates

Four different treatments before blood feeding were tested during 24h and 30h. Different solutions were provided to mosquitoes through a soaked cotton prior to blood feeding, including water only, a 10% sugar solution, a 3% salt solution and a total deprivation. As described before, 50 five to eight-day-old adult females from each *Ae. aegypti* Cayenne F1 strain were selected and placed in separate cages. Depending on the starvation duration, female mosquitoes were either immediately offered a blood meal for 30 min or starved for the specified period before being offered the blood meal for the same duration. All females were offered a blood meal using a 35mm-wide and 10 mm-deep Petri dish covered with Parafilm with the corresponding technical details as given in Table 1. Survival was monitored at the start of blood feeding. Experiment was performed in triplicate.

### Statistical analyses

All statistical analyses were performed using R 4.4.2, and RStudio Pro 2024.04.2. Data were cured and pre-processed in Microsoft Excel, version 16.43 before importing the data into R. All hypotheses were tested using generalized linear mixed models (GLMM), except for survival analyses where generalized linear models (GLM) were used. As the response variable for all models was either success (1) or failure (2), for either survival or blood-feeding rate, this was fitted to a binomial distribution. Models included the blood-feeding method, the *Aedes* strain, and their interaction as fixed effects, while replicate was included as random effect. These tests were ran using the *lme4* package [48] and figures were all made with the *base* package [49] and modified with Adobe Illustrator 2024. Data were analyzed by building a maximal model to test relevant variables and interactions. Likelihood Ratio Tests (LRT) from a maximal model were applied to assess the contribution of each fixed effect from the model using the *drop1* function from the *stats* package [49]. When categorical variables were significant, Post-Hoc Tukey tests analyses were conducted using *emmeans* and *contrast* functions from the *emmeans* package [50]. Full details of all data from our experiments and all the figures are presented in S1 Table. More details on statistical methods are provided in S1 File and S1 Code.

## Supporting information

S1 File Statistical analyses: details on the different steps for each statistical analysis. **Table A,** Estimated marginal means and 95% confidence intervals for the interaction between blood feeding technique (Treatment) and mosquito strain. Data was derived from a GLMM fitted with a binomial distribution and a random intercept for Replicate. These values represent predicted blood feeding probabilities and were used to generate the interaction plot in the main manuscript (related to Fig 1A). **Table B**, Post hoc pairwise comparisons (Tukey-adjusted) of blood feeding technique (Treatment) within each mosquito strain (Line). Data was based on the significant interaction term from the GLMM (maximal model). The table includes estimates, standard errors, z-ratios, and adjusted p-values. Related to Fig 1. **Table C,** Post hoc pairwise comparisons (Tukey-adjusted) for the effect of total starvation time (Treatment) on mosquito survival. Data was based on the GLM (Model 3) after addition of pseudo-observations to address complete separation. The table includes estimates, standard errors, z-ratios, and adjusted p-values. Related to Fig 2. **Table D,** Post hoc pairwise comparisons (Tukey-adjusted) of blood feeding probability across total starvation treatments (Treatment) within mosquito strains. Data was derived from a GLMM with a significant Treatment × Line interaction (Model 4). The table reports pairwise contrasts of estimated marginal means, with corresponding statistics and adjusted p-values. Related to Fig 2. **Table E,** Post hoc pairwise comparisons (Tukey-adjusted) of blood feeding probability across type of starvation (Treatment). The table reports pairwise contrasts of estimated marginal means, with corresponding statistics and adjusted p-values. Related to Fig 3. **Table F,** Post hoc pairwise comparisons (Tukey-adjusted) of blood feeding probability time of starvation in Cayenne F1 mosquitoes for sugar 10% (no starvation) and total deprivation. The table reports pairwise contrasts of estimated marginal means, with corresponding statistics and adjusted p-values. Related to Figs 2 and 3.
(DOCX)

S1 Table Raw data: Full details of all data from our experiments and all the figures. (XLSX)

S1 Fig Effect of time of total deprivation on blood-feeding success. Data from Figs 2 and 3 were compiled together: blood-feeding rate of all female mosquitoes that were not starved (0h) or totally deprived during 24h or 30h. Blue dots represent the mean predicted values from final models and yellow bars represent 95% confidence intervals; n = 40–55 females per condition per replicate; 6 replicates.
(TIF)

S1 Code R codes: Full details on R code used for our statistical analyses. (R)
