# Supplementary material for: Bridging laboratory and field research: Method adjustments to blood feed field-derived Aedes aegypti
Source: PLoS Negl Trop Dis. 2026 May 18;20(5):e0013339. doi: 10.1371/journal.pntd.0013339 (PMC13249168; doi:10.1371/journal.pntd.0013339)
Supplement: S1 File — Table A, Estimated marginal means and 95% confidence intervals for the interaction between blood feeding technique (Treatment) and mosquito strain. Data was derived from a GLMM fitted with a binomial distribution and a random intercept for Replicate. These values represent predicted blood feeding probabilities and were used to generate the interaction plot in the main manuscript (related to Fig 1A). Table B, Post hoc pairwise comparisons (Tukey-adjusted) of blood feeding technique (Treatment) within each mosquito strain (Line). Data was based on the significant interaction term from the GLMM (maximal model). The table includes estimates, standard errors, z-ratios, and adjusted p-values. Related to Fig 1. Table C, Post hoc pairwise comparisons (Tukey-adjusted) for the effect of total starvation time (Treatment) on mosquito survival. Data was based on the GLM (Model 3) after addition of pseudo-observations to address complete separation. The table includes estimates, standard errors, z-ratios, and adjusted p-values. Related to Fig 2. Table D, Post hoc pairwise comparisons (Tukey-adjusted) of blood feeding probability across total starvation treatments (Treatment) within mosquito strains. Data was derived from a GLMM with a significant Treatment × Line interaction (Model 4). The table reports pairwise contrasts of estimated marginal means, with corresponding statistics and adjusted p-values. Related to Fig 2. Table E, Post hoc pairwise comparisons (Tukey-adjusted) of blood feeding probability across type of starvation (Treatment). The table reports pairwise contrasts of estimated marginal means, with corresponding statistics and adjusted p-values. Related to Fig 3. Table F, Post hoc pairwise comparisons (Tukey-adjusted) of blood feeding probability time of starvation in Cayenne F1 mosquitoes for sugar 10% (no starvation) and total deprivation. The table reports pairwise contrasts of estimated marginal means, with corresponding statistics and adjusted p-values. Related [file pntd.0013339.s001.docx]

***Statistical analyses***

All statistical analyses were performed using R 4.4.2, and RStudio Pro 2024.04.2. Data were cured and pre-processed in Microsoft Excel, version 16.43 before importing the data into R. All hypotheses were tested using generalized linear mixed models (GLMM), except for survival rate analyses where generalized linear models (GLM) were used. These tests were ran using the lme4 package [1] and figures were all made with the base package [2] and edited in Adobe Illustrator 2024 for visual clarity.

To analyze the effect of the artificial blood-feeding method on the blood-feeding rate of the two Ae. aegypti strains, a maximal model was constructed. Here, blood-feeding rate was fitted as the response variable following a binomial distribution with an interaction of Ae. aegypti strain (two categories) and blood-feeding method (four categories) as fixed effects, while replicate (six replicates) was set as a random effect.

For the analyses to evaluate the preference of blood-feeding from mice or Hemotek devices, blood-feeding rate was fitted as the response variable following a binomial distribution. Blood-feeding source (two categories) was set as the fixed effect, while cage (14 cages) and replicate (two categories) were set as random effects.

The last analyses that focused on the efficiency of sugar starving before blood-feeding, survival rate and blood-feeding rate were analyzed. In both cases, the response variable was fitted with a binomial distribution, with Ae. aegypti strain (two categories), and sugar-starving time before blood-feeding (four categories) as fixed effects. In the model analyzing the blood-feeding rate, variable replicate (three replicates) was added as random effect.

In all analyses where blood-feeding rate was set as the response variable, this was set to 0 when individuals had not blood-fed and 1 when individuals were seen to be fully engorged. Where survival rate was set as the response variable, this was set to 0 when individuals died before the blood-feeding experiment and 1 when individuals were alive. For all models, Likelihood Ratio Tests (LRT) from a maximal model were applied to assess the contribution of each fixed effect from the model using the drop1 function from the stats package [2]. In order to assess the individual differences of the blood-feeding method within each of the two Ae. aegypti strains, Post-Hoc Tukey tests analyses were conducted using emmeans and contrast functions from the emmeans package [3].

Finally, the integrity of the spermathecae within each mosquito was analysed in function of Ae. aegypti strain, dissection buffer solution, and sampling condition (whether the mosquito was alive or had drowned before dissection). A full model was constructed with the response variable defined as the number of intact spermathecae per mosquito (out of 3 total), fitted using a binomial distribution. This model estimates the probability that a spermatheca remains intact, with each mosquito contributing three trials. In addition to the fixed effects mentioned, an interaction between sampling condition and Ae. aegypti strain was tested. The four replicates were included as a random effect. LRTs were applied to a maximal model to assess the individual contribution of each fixed effect.

**Table A. Estimated marginal means and 95% confidence intervals for the interaction between blood feeding technique (Treatment) and mosquito strain.** Data was derived from a GLMM fitted with a binomial distribution and a random intercept for Replicate. These values represent predicted blood feeding probabilities and were used to generate the interaction plot in the main manuscript (related to figure 1A)

| **Treatment** | **Strain** | **Fitted mean** | **se** | **Lower CI** | **Upper CI** |
| --- | --- | --- | --- | --- | --- |
| Collective lids | New Orleans | 0.77 | 0.05 | 0.65 | 0.86 |
| Hemotek 3mL | New Orleans | 0.64 | 0.06 | 0.51 | 0.75 |
| Individual lids | New Orleans | 0.8 | 0.05 | 0.68 | 0.87 |
| Petri 4mL | New Orleans | 0.7 | 0.06 | 0.57 | 0.8 |
| Collective lids | Wild Cayenne F1 | 0.3 | 0.06 | 0.2 | 0.42 |
| Hemotek 3mL | Wild Cayenne F1 | 0.24 | 0.05 | 0.15 | 0.36 |
| Individual lids | Wild Cayenne F1 | 0.59 | 0.07 | 0.45 | 0.71 |
| Petri 4mL | Wild Cayenne F1 | 0.21 | 0.05 | 0.13 | 0.32 |

**Table** **B. Post hoc pairwise comparisons (Tukey-adjusted) of blood feeding technique (Treatment) within each mosquito strain (Line**). Data was based on the significant interaction term from the GLMM (maximal model). The table includes estimates, standard errors, z-ratios, and adjusted p-values. Related to figure 1.

| **Pairwise comparisons** | **Line** | **Estimate** | **SE** | **Z-ratio** | ***p-*value** |
| --- | --- | --- | --- | --- | --- |
| Collective lids - Hemotek 3mL | New Orleans | 0.63 | 0.26 | 2.42 | 0.07 |
| Collective lids - Individual lids | New Orleans | -0.15 | 0.28 | -0.52 | 0.96 |
| Collective lids - Petri 4mL | New Orleans | 0.37 | 0.27 | 1.4 | 0.5 |
| Hemotek 3mL - Individual lids | New Orleans | -0.78 | 0.27 | -2.89 | 0.02 |
| Hemotek 3mL - Petri 4mL | New Orleans | -0.26 | 0.25 | -1.04 | 0.73 |
| Individual lids - Petri 4mL | New Orleans | 0.52 | 0.27 | 1.89 | 0.23 |
| Collective lids - Hemotek 3mL | Cayenne F1 | 0.27 | 0.26 | 1.04 | 0.72 |
| Collective lids - Individual lids | Cayenne F1 | -1.21 | 0.25 | -4.91 | <0.001 |
| Collective lids - Petri 4mL | Cayenne F1 | 0.46 | 0.27 | 1.69 | 0.33 |
| Hemotek 3mL - Individual lids | Cayenne F1 | -1.49 | 0.26 | -5.82 | <0.001 |
| Hemotek 3mL - Petri 4mL | Cayenne F1 | 0.18 | 0.28 | 0.66 | 0.91 |
| Individual lids - Petri 4mL | Cayenne F1 | 1.67 | 0.26 | 6.34 | <0.001 |

**Table C. Post hoc pairwise comparisons (Tukey-adjusted) for the effect of total starvation time (Treatment) on mosquito survival**. Data was based on the GLM (Model 3) after addition of pseudo-observations to address complete separation. The table includes estimates, standard errors, z-ratios, and adjusted p-values. Related to figure 2.

| **Pairwise comparisons** | **Estimate** | **SE** | **Z-ratio** | ***p-*value** |
| --- | --- | --- | --- | --- |
| Treatment 0 – Treatment 24 | 0.89 | 0.5 | 1.79 | 0.28 |
| Treatment 0 – Treatment 30 | 1.29 | 0.47 | 2.74 | 0.03 |
| Treatment 0 – Treatment 48 | 2.93 | 0.43 | 6.74 | <0.001 |
| Treatment 24 – Treatment 30 | 0.4 | 0.36 | 1.13 | 0.67 |
| Treatment 24 – Treatment 48 | 2.04 | 0.31 | 6.66 | <0.001 |
| Treatment 30 – Treatment 48 | 1.64 | 0.26 | 6.19 | <0.001 |

**Table D. Post hoc pairwise comparisons (Tukey-adjusted) of blood feeding probability across total starvation treatments (Treatment) within mosquito strains**. Data was derived from a GLMM with a significant Treatment × Line interaction (Model 4). The table reports pairwise contrasts of estimated marginal means, with corresponding statistics and adjusted p-values. Related to figure 2.

| **Pairwise comparisons (hours of starvation)** | **Strain** | **estimate** | **SE** | **Z- ratio** | ***p-*value** |
| --- | --- | --- | --- | --- | --- |
| 0 - 24 | New Orleans | 0.18 | 0.25 | 0.73 | 0.89 |
| 0 - 30 | New Orleans | -0.01 | 0.25 | -0.04 | 1 |
| 0 - 48 | New Orleans | 0.25 | 0.27 | 0.92 | 0.79 |
| 24 - 30 | New Orleans | -0.19 | 0.25 | -0.76 | 0.87 |
| 24 - 48 | New Orleans | 0.07 | 0.27 | 0.27 | 0.99 |
| 30 - 48 | New Orleans | 0.26 | 0.28 | 0.95 | 0.78 |
| 0 - 24 | Cayenne F1 | 0.36 | 0.4 | 0.9 | 0.81 |
| 0 - 30 | Cayenne F1 | -0.98 | 0.32 | -3.02 | 0.01 |
| 0 - 48 | Cayenne F1 | -0.95 | 0.34 | -2.76 | 0.03 |
| 24 - 30 | Cayenne F1 | -1.33 | 0.36 | -3.71 | <0.001 |
| 24 - 48 | Cayenne F1 | -1.3 | 0.38 | -3.46 | <0.001 |
| 30 - 48 | Cayenne F1 | 0.03 | 0.3 | 0.09 | 1 |

**Table E. Post hoc pairwise comparisons (Tukey-adjusted) of blood feeding probability across type of starvation (Treatment)**. The table reports pairwise contrasts of estimated marginal means, with corresponding statistics and adjusted p-values. Related to figure 3.

| **Pairwise comparisons (hours of starvation)** | **Strain** | **estimate** | **Z- ratio** | ***p-*value** |
| --- | --- | --- | --- | --- |
| H2O - NaCl 3% | -0.96 | 0.21 | -4.47 | <0.0001 |
| H2O - Sugar 10% | 0.03 | 0.24 | 0.13 | 0.9992 |
| H2O - Total deprivation | -1.06 | 0.21 | -5.0 | <0.0001 |
| NaCl 3% - Sugar 10% | 0.99 | 0.21 | 4.63 | <0.0001 |
| NaCl 3% - Total deprivation | -0.1 | 0.18 | -0.56 | 0.94 |
| Sugar 10% - Total deprivation | -1.09 | 0.21 | -5.15 | <0.0001 |

**Table F. Post hoc pairwise comparisons (Tukey-adjusted) of blood feeding probability time of starvation in Cayenne F1 mosquitoes for sugar 10% (no starvation) and total deprivation**. The table reports pairwise contrasts of estimated marginal means, with corresponding statistics and adjusted p-values. Related to figure 2 and 3.

| **Pairwise comparisons (hours of starvation)** | **Strain** | **estimate** | **Z- ratio** | ***p-*value** |
| --- | --- | --- | --- | --- |
| 0 h – 24 h | -0.4481 | 0.2123 | -2.1105 | 0.0877 |
| 0 h – 30 h | -1.197 | 0.1964 | -6.0952 | 0 |
| 24 h – 30 h | -0.7489 | 0.2022 | -3.7034 | 6,00E-04 |

**References:**

1. Bates, D.; Mächler, M.; Bolker, B.; Walker, S. Fitting Linear Mixed-Effects Models Using Lme4. *Journal of Statistical Software* **2015**, *67*, 1–48, doi:10.18637/jss.v067.i01.

2. R Core team (2024) R: A Language and Environment for Statistical Computing. R Foundation for Statistical Computing, Vienna Available online: https://www.R-project.org/.

3. Lenth (2024), R.V. Emmeans: Estimated Marginal Means, Aka Least-Squares Means Available online: {https://rvlenth.github.io/emmeans/}.
