# Supplementary material for: Bridging laboratory and field research: Method adjustments to blood feed field-derived Aedes aegypti
Source: PLoS Negl Trop Dis. 2026 May 18;20(5):e0013339. doi: 10.1371/journal.pntd.0013339 (PMC13249168; doi:10.1371/journal.pntd.0013339)
Supplement: Attachment — Submitted filename: review..docx [file pntd.0013339.s005.docx]

Dear Authors,

Below are my reviews.

1. While this abstract provides a clear introduction to the subject matter and explains the importance of the problem, it falls short by not presenting the study’s results. A well-structured abstract should summarize not only the background and rationale but also the main findings. As a reader, I want to understand the key outcomes of the study from the abstract alone so I can decide whether to read the full paper. Without results, the abstract feels incomplete and limits its usefulness.
2. The Author Summary introduces the study topic, explains its importance and even describes the methods used, but it does not present the main findings. This section should summarize the entire study in accessible language, including the key results and their implications, so that readers can understand what was discovered and why it matters without having to read the full manuscript.
3. I was not able to clearly identify the main objective of the study in either the abstract or the Author Summary, but it is explicitly stated in the introduction. While it may be implied, it does not come across explicitly. Both sections should state the study’s primary goal in straightforward terms so that readers can immediately understand the focus of the work without needing to read the full manuscript,
4. The meaning of “Ae. aegypti” should be defined upon its first use in the abstract (e.g., “Aedes aegypti (Ae. aegypti)”) and then used consistently throughout the text. This ensures clarity for readers who may not be familiar with the abbreviation and aligns with standard taxonomic conventions.
5. While I appreciate the detailed supporting document that outlines the statistical models used, the manuscript would greatly benefit from including the underlying raw data. Specifically, it would be helpful to provide the individual mosquito counts, including the number tested, those that successfully fed, those that died, and etc. Making these data available would strengthen reproducibility and transparency, allowing other researchers to verify the results or reanalyze the findings using alternative approaches.
6. Overall, this is a well-written and carefully executed study. The findings will make a valuable contribution to the field and will help inform future research on vector competence, as well as guide both laboratory and field-based studies involving Aedes aegypti and other mosquito species.
