# Supplementary material for: Bridging laboratory and field research: Method adjustments to blood feed field-derived Aedes aegypti
Source: PLoS Negl Trop Dis. 2026 May 18;20(5):e0013339. doi: 10.1371/journal.pntd.0013339 (PMC13249168; doi:10.1371/journal.pntd.0013339)
Supplement: Attachment — Submitted filename: Response to reviewers.docx [file pntd.0013339.s007.docx]

**Additional Editor comments:**

Thank you for your submission to PLoS NTDs and my apologies for the delay in getting feedback on your manuscript to you. The reviewers all felt that your manuscript presenting studies on work with field versus lab mosquitoes is valuable, relevant and overall well-executed. However, there are aspects of the manuscript that require revision to improve clarity/cohesion, justification of experimental design decisions, and thoroughness in reporting on methods and sample sizes. No additional experimental work is requested, but the reviewers recommend revisions to clarify these aspects.

Specifically, the reviewers would like you to address the following:

The hypotheses behind the different aspects of the study are present, but not always clearly articulated and need to be more explicitly stated

The introduction does not set up the rationale or relationship between the experiments. For example, why were spermathecae integrity and dissection media were tested along with feeding assays?

Thank you for this relevant comment. Overall, this study aims to put together some method adjustments that we decide to do when working with field-derived mosquitoes in general; this included blood feeding experiments and spermatheca dissections, and even though the latter eventually did not show any difference between strains, we decided to keep these results to guide other researchers in their experimental designs. We now added a paragraph to the introduction to better frame spermatheca dissections:

“Mosquito reproduction depends on physiological and behavioral processes in fertilized females. In Ae. aegypti, these processes include host-seeking behavior leading to increased blood-feeding behaviors, modifications in egg development, and sperm storage [25]. Furthermore, female Ae. aegypti typically mate only once, so the storage of sperm in the spermathecae is crucial to optimize reproductive success [26]. The spermathecal environment must be conducive to maintaining sperm viability until their release for egg fertilization [27]. When rearing mosquitoes, it is sometimes required to dissect spermatheca to monitor the presence of sperm therein as a readout of female fertilization. Various isotonic solutions have been used for this purpose such as Aedes saline solution [28], Ringer's solution [29], and phosphate-buffered saline (PBS) [30].

We also add a sentence to clarify the test concerning starvation period.

The relevance/significance of the experiments should be clarified in the introduction rather than the results.

Thanks

We now clarified this.

Additional clarity on the materials and methods is needed. Specifically:

Samples sizes on the spermathecae experiments need to be reported.

Thank you for this comment, we completed the methodology section concerning spermathecae. Please note that for a sake of reproducibility, considering the reviewers comments, we did the spermatheca study again.

Methods now read as follows:

“Female mosquitoes were collected from cages where they had spent between 4 and 8 days with males. Two scenarios were tested: the dissection of females collected alive, and the dissection of females that were intentionally drown in water for 24h.Live females were briefly frozen before being dissected under a stereomicroscope in a drop of buffer solution. The last abdominal segment was pulled apart and opened with 0.2 mm Minutien pins mounted on pin holders to remove the spermathecae. Integrity of the three spermathecae and presence of spermatozoa were then observed under a phase-contrast microscope with a magnification of 100X. For each strain, buffer solution and experiment, 10 females were sampled in triplicate.”

Description of the rationale behind the use of different feeding methodologies.

We agree with this comment and in the methods, we now explain the use of different feeding methodologies:

“These methods were selected for several reasons. Firstly, the Hemotek system, which is a standard method for feeding mosquitoes used in many insectaries worldwide, allows for precise control of blood temperature and is typically used with a collagen membrane. However, it is limited when it comes to conducting multiple feedings simultaneously or working in laboratories with scarce equipment. In comparison, we chose Petri dishes along with Parafilm, as they are accessible and inexpensive in laboratory settings. Microtube lids, very common laboratory consumables, were used to test methods for monitoring individual mosquitoes. Using several caps on large cages allowed for a comparison with the two other methods on cages described above.”

Clarification of the rationale behind starvation times.

These time periods were chosen both because they represent a standard method and to facilitate conducting blood feeds at two different times in a typical working day. This point has been clarified in the methods:

“The different deprivation periods were selected based on the literature (typically 24 h) and to accommodate feeding the mosquitoes at convenient timings in the morning and the afternoon.”

Moreover, we added a new experiment concerning different treatments before blood feeding that is explained in the manuscript:

See in the methods:

“**Impact of deprivation treatments on blood feeding rates**

Four different treatments before blood feeding were tested during 24h and 30h. Different solutions were provided to mosquitoes through a soaked cotton prior to blood feeding, including water only, a 10% sugar solution, a 3% salt solution and a total deprivation. As described before, 50 five to eight-day-old adult females from each Ae. Aegypti Cayenne F1 strain were selected and placed in separate cages. Depending on the starvation duration, female mosquitoes were either immediately offered a blood meal for 30 min or starved for the specified period before being offered the blood meal for the same duration. All females were offered a blood meal using a 35mm-wide and 10 mm-deep Petri dish covered with Parafilm with the corresponding technical details as given in Table 1. Survival was monitored at the start of blood feeding. Experiment was performed in triplicate.”

See in the results:

“We further tested whether hunger or thirst was the essential driver to blood feeding, providing mosquitoes with water only or with a hyperosmotic aqueous solution containing 3% NaCl, keeping 10% sugar solution and total deprivation as non-starved and fully starved controls. In this experiment, starved and non-starved mosquitoes were proposed a blood-meal at the same time to account for potential differences due to circadian-rhythm related issues. We selected relevant conditions from the previous experiment, focusing on Cayenne F1 mosquitoes and on 24h and 30h timings.

We found that starvation time had a significant impact on mortality (X2(1) =4.21, p=0.040), but that mortality remained low at 30h (0.062 ± 0.014). Starvation type and time both significantly affected blood feeding rates (starvation type: X2(3) = 49.65, p<0.001; time: (X2(1) =5.90, p=0.015; Figure 3). As there was no significant interaction between starvation type and time, we performed post-hoc analyses starvation types, pooling mosquitoes from both time points. We found that mosquitoes provided with water only did not feed better than non-starved mosquitoes (p=1.0), while total deprivation and feeding with salt solution significantly affected blood feeding rate (p<0.001) to a similar extent (total deprivation vs NaCl solution: p=0.94; see Table E in S1 text for more details). This indicates that thirst rather than lack of energy drives attraction to blood feeding, at least until 30h.

Considering starvation timing, we observed a slight discrepancy between Figures 2 and 3 on the impact of total deprivation at 24h. Compiling together our data from Cayenne F1 that were not starved or totally deprived during 24h or 30h, we found that a 30h starvation highly significantly affected blood feeding rate (X2(2)=38,89, p<0.0001) while a 24h starvation had a marginally significant impact (p=0.088; Supplementary figure S1; data from Figures 2 and 3). The difference between a 24h and a 30h starvation was strongly significant (p=0.0006). To account for potential effects of circadian rhythms, we tested whether non-starved mosquitoes fed in the morning (9 am for 24 h) and afternoon (3 pm for 30 h) had a different blood feeding rate (data from Figure 3). This schedule did not significantly affect feeding rate (X2(1)=1.79, p=0.18), suggesting that the difference in feeding rate between 24h and 30h starvation was largely linked to prolongation of starvation itself.”

See in the discussion:

“Comparing different types of starvation, we showed that thirst rather than lack of energy was the main driver for mosquito attraction. Indeed, providing water only completely abrogated the impact of starvation. On the contrary, mosquitoes provided a hyperosmotic NaCl solution, with a salt concentration close to sea water, were as attracted to blood as completely starved mosquitoes. The strong differences observed between types of starvation highlight the importance of specifying how starvation is conducted in scientific manuscripts. For instance, indications such as “sugar starvation” or “sucrose deprivation” require clarification. The results observed with salt water are intriguing; we are not sure that mosquitoes indeed ingested the 3% salt solution and became thirsty via a “crisps and peanut effect” or if they were just water deprived. Yet, we observed mosquitoes on the cotton. The presence of salt in water (1%) has been reported not to affect mosquito survival, whether in the presence or absence of sugar [34]. This suggests that salt water, or potentially water containing salt and sugar, may allow to starve mosquitoes while avoiding death by desiccation. Further work would be required to validate this and to check that, for each experimental set up, salt does not act as a confounding factor.”

Details on experiments need to be included including blood temperatures and timing on activation of hand warmers.

We took into account your comment and added in the methods some details about hand warmers:

“Except for Hemotek that had its own electronic heating system at 37°C, all other treatments were kept warm with gel hand warmers of sodium acetate that were left on top of the blood-feeding devices after getting activated. The hand warmers activate within seconds, reaching 39 ± 1°C and maintaining this temperature throughout the 30min blood meal. Unlike the metal capsule of the Hemotek system that allows the blood to reach a temperature of 37°C within 3 minutes, when using a plastic Petri dish or a tube lid, the blood reaches a temperature of 36°C within about 10 minutes and 3 minutes respectively.”

Clarification on if the mosquitoes were F1 progeny or directly from field collections.

Thank you, we clarified this point, adding that mosquitoes that we collected as larvae or pupae are considered as the parental generation. We then worked with their offspring, which constituted the F1 mosquito generation.

Presentation of the results could be improved:

- Sample sizes and the number of replicates should be included.

We prepared a Supplementary Table S1 that includes the number of sampled mosquitoes and replicates for each experiment.

- The Y-axis in figure 3 "expected proportion of intact spermathecae" is unclear and should be revised for clarity

We now clarified this in figure 4 (ex-figure 3) with y axis labelled “Proportion of females with intact spermathecae” instead.

- Blood source comparisions in figure 1 should be clarified.

We now added bars indicating the most relevant statistical tests. We agree that it clarifies our message.

- There is an inconsistency between the feeding time listed in Figure 1 (45 mins) versus that described in the materials and methods (30 mins)

Sorry for this mistake in the figure 1. Our feeding time was 30min and we corrected it in the text.

Please include the raw data such as the number of mosquitoes tested, fed, observed mortality, for reproducibility purposes.

We prepared a Supplementary Table S1 that includes the number of sampled mosquitoes and replicates for each experiment. We also added number of mosquitoes and replicates in the legend of each figure.

Over statement/interpretation of conclusions:

- Discussion on the limitations of using a single field strain and consideration of variability between genetically distinct strains would be helpful.
- More discussion on the differences between colony and near-field mosquitoes in situations such as sugar starvation.

We add a pararagraph concerning these points in the discussion as follows:

“Vector biology experiments range from basic research on reference strains to applications such as the release of mosquitoes or the implementation of vector control strategies. Between these extremes, a whole set of intermediates set ups exist and are being developed to ensure a step-by-step path from discovery to application. This includes the use of field-derived mosquitoes, variable microbial set-ups, diverse types of cage sizes or other aspects of mosquito husbandry [46,54]. In this context, our study provides valuable methodological adjustments to work with field-derived Ae. aegypti. We used Cayenne F1 as a model, which derives from several collections (one per replicate) in the town of Cayenne. Local adaptation and genetic variability between distinct field populations can however influence physiological and behavioral traits [46], so the observed differences between New Orleans and Cayenne F1 may not be fully generalizable to other field populations. Overall, our results may either be used as such, using the parameters that we found most relevant to study field-derived mosquitoes, or as a framework for method improvement, as they show some important factors affecting the quality of fitness studies.”

We also add some details in the paragraph concerning starvation:

“For New Orleans mosquitoes, our data indicates that starvation is unnecessary and that feeding techniques have a minimal impact. This indicates that New Orleans strain has undergone adaptation to laboratory conditions, enabling more efficient feeding through artificial membranes without requiring additional stimuli such as starvation. This may not be surprising as laboratory environments exert different selective pressure compared to field environments, as laboratory diet is abundant, temperature and humidity are relatively controlled [10], yet artificial feeding lacks natural stimuli and the microbiota may be unbalanced [46,47]. While this effect is detectable with Ae aegypti, some traits have been surprisingly well conserved over the years, such as circadian peaks of activity [44,45] and resistance to starvation (as shown here). Considering the anthropophilic behavior and its tendency to breed in artificial containers, we can assume that other mosquito species may undergo even more stringent selection during colonization.”

Statements about environmental factors being "impossible" to study in the field should be softened.

Ok, we replaced “impossible” with “extremely complex”.

Clarification of why unburst spermathecae are of interest when microscopic examination of burst spermathecae is standard for fertiliy status assessment.

This point from reviewer 3 is completely correct.

To verifying mosquito fertilization—particularly when assessing a potential influence on the blood meal—the parameter of interest is typically the presence of sperm in at least one of the three spermathecae. During routine examination, observing an intact spermatheca facilitates this assessment, but it is indeed not necessary. Actually, spermathecae were chosen as a model tissue—not only for their link to mosquito fertility but—to verify whether Cayenne field mosquitoes differ from laboratory mosquitoes during dissections. The rationale for this choice is simply that a difference was initially observed during such dissections between different media used with Cayenne mosquitoes but not with the New Orleans strain. After replication in more standard conditions however, we did not observe any difference. We think that it is interesting for researchers to know that these buffers do not differ so that they can use the most convenient for them. We however observed difference when mosquiotes were drown in water for 24h.

We added some explanation in the results:

“*Ae. aegypti* females are typically fertilized during their first mating and then carry spermatozoa in their spermatheca. When rearing mosquitoes, it can also be useful to quantify fertilization, monitoring the presence of sperm cells in dissected spermatheca. As spermathecae sometimes burst during dissection, we used this type of dissection as a readout to account for tissue integrity along the experiment according to mosquito strain and dissection buffer. Two solutions were compared: PBS and *Aedes* saline, which was formulated to provide optimal osmotic pressure for mosquito dissections [31]. Assessing fertilization is typically interesting when a colony is struggling, and it is necessary to determine why females are not laying eggs. In such cases, it can be useful to also sample females shortly after death, as live females are sometimes too valuable to be collected. Mosquitoes that fall on the bottom of the cage dry out quickly, but those that drown in water can often be dissected several hours later. Therefore, we also tested whether mosquitoes that had drowned 24h beforehand could still be dissected successfully (Figure 4A).”

Expanded discussion on the broader implications for field and lab practices given your findings.

In the discussion we added a paragraph concerning the differences between lab and semi-field mosquitoes.

See in the discussion:

“Local adaptation and genetic variability between distinct field populations can however influence physiological and behavioral traits [46], so the observed differences between New Orleans and Cayenne F1 may not be fully generalizable to other field populations. Overall, our results may either be used as such, using the parameters that we found most relevant to study field-derived mosquitoes, or as a framework for method improvement, as they show some important factors affecting the quality of fitness studies. Beyond the adjustments proposed here, other experimental set ups can improve work with field mosquitoes. It is preferable to increase the number of mosquitoes collected in the field and to provide them ideal conditions, notably recurrent blood feedings with several blood sources to obtain a large progeny [52]. These adjustments may be useful for colonization and for studies on vector competence or insecticide resistance, which require a large number of mosquitoes.”

There were also a number of stylistic revisions recommended:

Make sure to include the main findings and explicitly state the studies goals in the abstract and author summary.

We agree and added some missing points in the abstract as follows:

“ In this study, we evaluated methods to manipulate field-derived mosquitoes from Cayenne (French Guiana), using the New Orleans reference strain as a control. To improve blood feeding rate, we tested the impact of several blood feeding systems for mosquitoes kept in a cage or in individual vials and adjusted starvation parameters. Individual mosquitoes offered a bloodmeal in lids of microcentrifuge tubes had the highest blood feeding rate. For field-derived mosquitoes, starvation, or alternatively provision of a 3% salt solution instead of the sugar solution, also consistently improved blood feeding rate, with a minimal impact on survival. We also assessed whether the dissection buffer needed any adjustment for field derived mosquitoes, using spermatheca integrity as a read-out. We found no difference between mosquito strains or buffer, but observed that spermatheca of mosquitoes drown for 24h can still be dissected. In conclusion, our results may be helpful for establishment of new colonies, including in resource-limited settings, studies on fitness of field-derived mosquitoes and on experiments requiring individual level monitoring.”

And in the author summary:

“In this study, we adjusted methods to manipulate field-derived mosquitoes from Cayenne (French Guiana), using the New Orleans reference strain as a control. To improve blood feeding rate, we tested the impact of several blood feeding systems for mosquitoes kept in a cage or in individual vials and adjusted starvation conditions. Our data indicate that the reference strain blood feeds relatively well and is not much impacted by experimental adjustments, while blood feeding of field-derived mosquitoes is substantially affected by blood container and starvation. We also tested differences in dissection using spermatheca integrity as a readout, and did not find any relevance in modifying dissection buffer. Interestingly, spermatheca can still be dissected 24h after mosquitoes drowning, which may be interesting for colony maintenance without sacrificing any female. Our results may enhance experimental reliability of colony establishment and maintenance, including in resource-limited settings and of studies on the fitness of field-derived mosquitoes, including at the individual level.”

Introduce Aedes aegypti in full at its first mention and abbreviate later mentions.

Thank you for this comment, we have corrected this.

Make sure terminology is consistent. For example: "beef blood" versus "bovine blood"; "survival" versus "survival rate".

Thank you for this. We changed the words to make it more consistent throughout the manuscript.

**Reviewers' Comments:**

Reviewer's Responses to Questions

**Key Review Criteria Required for Acceptance?**

As you describe the new analyses required for acceptance, please consider the following:

**Methods:**

-Are the objectives of the study clearly articulated with a clear testable hypothesis stated?

-Is the study design appropriate to address the stated objectives?

-Is the population clearly described and appropriate for the hypothesis being tested?

-Is the sample size sufficient to ensure adequate power to address the hypothesis being tested?

-Were correct statistical analysis used to support conclusions?

-Are there concerns about ethical or regulatory requirements being met?

Reviewer #1: - The hypotheses are present, though not always clearly stated.
- The study design, overall, appears appropriate

- The populations are clearly defined. It would be ideal to have an established, long-term colony of local mosquitoes as control, but the New Orleans strain is likely a good proxy as a regional, relatively recently derived strain

When dealing with long-term colonies, we prefer using a reference strain (New Orleans) to reduce differences between labs, and then we validate some tests using mosquitoes that are more directly field derived.

- It is unclear if the sample size is sufficient. Sample size for the spermathecae experiments does not appear to be reported. Thus, the difference found, though significant, is unclear if it should be dismissed as an artifact of low sample size or if it's a true difference.

We add a supplementary table with the raw data to make it clearer. Moreover, more spermathecae were dissected in order to obtain a sufficient sample size for statistical analyses.

- Suitable statistical tests were used, though it would have also have been suitable to perform non model-based statistics with the appropriate classical statistical checks. This might improve interpretation by a broader audience. It was nice to see the experimental conditions included as random effects, though, as an additional check.

We don’t think that is necessary, but we now provide a supplementary R code file to clarify the choices of the different statistical tests that were used.

- No ethical or regulatory concerns were identified.

In the methodology section, we specified the past use of mice, particularly for maintaining mosquito colonies.

“For Figure 1B and some blood meals of New Orleans colony maintenance, anaesthetized mice have been used for mosquito blood feeding. Protocols have been validated by the French Direction générale de la recherche et de l’innovation, ethical board # 089, under the agreement # 973021.”

Reviewer #2: yes

Reviewer #3: (No Response)

**Results**


-Does the analysis presented match the analysis plan?

-Are the results clearly and completely presented?

-Are the figures (Tables, Images) of sufficient quality for clarity?

Reviewer #1: -

The analysis matches the analysis plan

- The results are completely presented, however clarity is limited by words and turns of phrase covered in the Methods section that is not until later.

We hope that the reviewer will be convinced by the clarifications throughout the text. We decided to lead the reader through the rationale of the study in the results before getting specific experimental details in the methods.

- Figure 1 states that mosquitoes were fed for 45 minutes, while the Methods section states 30 minutes.

Corrected. We apologize for this mistake.

Reviewer #2: yes

Reviewer #3: (No Response)

**Conclusions:**

-Are the conclusions supported by the data presented?

-Are the limitations of analysis clearly described?

-Do the authors discuss how these data can be helpful to advance our understanding of the topic under study?
-Is public health relevance addressed?

Reviewer #1: - The conclusions are supported by the data presented (with the caveat of the missing sample size previously mentioned).

- Limitations not discussed include not using a colony of locally-derived Ae. aegypti (though maybe not practical for this study, others have show significant behavioral and genetic differences in populations of this species within a single country over relatively short regional distances);

We expanded our discussion as described in editor comments.

" Vector biology experiments range from basic research on reference strains to applications such as the release of mosquitoes or the implementation of vector control strategies. Between these extremes, a whole set of intermediates set ups exist and are being developed to ensure a step-by-step path from discovery to application. This includes the use of field-derived mosquitoes, variable microbial set-ups, diverse types of cage sizes or other aspects of mosquito husbandry [46,54]. In this context, our study provides valuable methodological adjustments to work with field-derived Ae. aegypti. We used Cayenne F1 as a model, which derives from several collections (one per replicate) in the town of Cayenne. Local adaptation and genetic variability between distinct field populations can however influence physiological and behavioral traits [46], so the observed differences between New Orleans and Cayenne F1 may not be fully generalizable to other field populations. Overall, our results may either be used as such, using the parameters that we found most relevant to study field-derived mosquitoes, or as a framework for method improvement, as they show some important factors affecting the quality of fitness studies.

Beyond the adjustments proposed here, other experimental set ups can improve work with field mosquitoes. It is preferable to increase the number of mosquitoes collected in the field and to provide them ideal conditions, notably recurrent blood feedings with several blood sources to obtain a large progeny [52]. These adjustments may be useful for colonization and for studies on vector competence or insecticide resistance, which require a large number of mosquitoes.”

the potential that the buffer/spermatheca issue could be a small sample size or sampling issue (i.e. no physiological or other basis for this difference is hypothesized, so it reads like an experimental artifact that seemed odd, so it made it into the paper);

Thanks for this point. We were also worried about this and tested it again by increasing the number of females and indeed do not detect any significant difference between dissection buffers anymore.

the limitation of different membranes for different feeders is not discussed (we use Parafilm membranes on Hemoteks without issue, so it is unclear as to why this extra variability was added);

Our experience does indeed lack the use of Hemotek capsules with parafilm, which seems to be a promising intermediary. In our laboratory, blood meals with Hemotek were performed exclusively collagen membrane, so when starting to use petri dishes, we also changed the membrane to parafilm as a sake of convenience. We are aware that this introduces additional variability, but we are comparing methods rather than each parameter of each method.

and a discussion point about near-field vs. colony mosquitoes and sugar starvation is missing (i.e. colony mosquitoes are reared with ad libitum access to sucrose while near-field mosquitoes are not used to having that access. There are likely physiological differences associated with this that help explain why the colony mosquitoes cannot tolerate extended sugar starvation in the way that the F1 colonies can.

Thank you for this interesting comment that we used to improve our discussion as follows:

“For New Orleans mosquitoes, our data indicates that starvation is unnecessary and that feeding techniques have a minimal impact. This indicates that New Orleans strain has undergone adaptation to laboratory conditions, enabling more efficient feeding through artificial membranes without requiring additional stimuli such as starvation. This may not be surprising as laboratory environments exert different selective pressure compared to field environments, as laboratory diet is abundant, temperature and humidity are relatively controlled [10], yet artificial feeding lacks natural stimuli and the microbiota may be unbalanced [46,47]. While this effect is detectable with Ae aegypti, some traits have been surprisingly well conserved over the years, such as circadian peaks of activity [44,45] and resistance to starvation (as shown here). Considering the anthropophilic behavior and its tendency to breed in artificial containers, we can assume that other mosquito species may undergo even more stringent selection during colonization.”

While the timing of the meals are a common critique, in our hands this species readily feeds in the field and in the lab at all hours at relatively consistent levels. So, I would encourage the authors to think more about why that difference may have shown up as it is interesting).

Thanks for this comment. We have been deeper in the discussion concerning bloodfeeding:

See in the discussion:

“We found that a 30h starvation significantly increased blood-feeding rate of Cayenne F1 from 0.11 to 0.30 (Figure S1), with a minimal effect on mosquito survival (0.045). Previous studies have shown that sugar starving of 6 to 24 h improves blood-feeding rates in Aedes or Anopheles [32,35–37]. Conversely, other traits such as host-seeking behavior, appear unaffected by sugar deprivation in female Ae. aegypti mosquitoes [24]. Our data suggest that the difference observed after 24h and 30h of starvation is not significantly linked to circadian rhythms of the mosquito according to the feeding rate of non-starved mosquitoes. While Ae. aegypti is overall diurnal [38,39], its activity patterns in the field are bimodal, with peak activity occurring at 7:00-8:00 a.m. and 7:00-8:00 p.m. [40–43]. Recent studies on laboratory-reared mosquitoes suggested a relatively strong feeding throughout the day, with a drop during ~3 h in the middle of the day that is driven by the mosquito internal clock [44,45]. Both times tested (9:00-9:30 a.m. and 3:00-3:30 p.m.) fall outside these highs and lows in activity, which may explain why we did not detect any time-related difference.”

- Public health relevance is addressed in the Discussion, though more could be said.

In the Discussion, we note that our findings are relevant for vector competence studies requiring comparison of field and reference strains, as well as for insecticide testing.

Reviewer #2: yes

Reviewer #3: (No Response)

**Summary and General Comments:**

Use this section to provide overall comments, discuss strengths/weaknesses of the study, novelty, significance, general execution and scholarship. You may also include additional comments for the author, including concerns about dual publication, research ethics, or publication ethics. If requesting major revision, please articulate the new experiments that are needed.

Reviewer #1: The authors put forth several connected studies that investigate the differences between their local F1 Ae. aegypti and a standardized colony strain. Consistent production of quality, high number near-field material is important to the field, and any tools and protocols that can help further that work are certainly welcome.

The authors have clearly put forth a great deal of effort into compiling these studies and this manuscript and should be commended. I believe the previous comments cover the major points of concern. If there are justifications for the use of different membranes and the sample size for the spermatheca dissections was sufficient and seems to be a true difference with some plausible explanation, then I do not believe that any experiments need to be repeated to major revisions undertaken.

I believe the strength of this manuscript lies in the feeding success rates, sugar starvation times, and feeding devices. A manuscript with more detail on those points demonstrating a reproducible and reliable method for bringing in and generating F1s would be very helpful to the field.

Comments on blood meals, desiccation, and a more detailed explanation of our data have been added throughout the manuscript to address this point.

Reviewer #2: Overall, this is a well-written manuscript that addresses an important research problem involving mosquito studies in low-cost settings. The study is relevant and will provide valuable insights for future work on vector competence and related laboratory and field studies.

That said, I identified several areas that would benefit from revision. First, the abstract and author summary are incomplete, as they do not include the main findings of the study. Both sections currently introduce the topic and highlight its importance but fail to communicate the results, which are essential for readers to quickly grasp the contribution and impact of the work. Second, the main goal of the study is not clearly and explicitly stated in either the abstract or the author summary. While it may be implied, it should be presented in straightforward terms so that readers immediately understand the central objective of the work.

We previously received this comment and have now addressed it accordingly.

See in the abstract:

“ In this study, we evaluated methods to manipulate field-derived mosquitoes from Cayenne (French Guiana), using the New Orleans reference strain as a control. To improve blood feeding rate, we tested the impact of several blood feeding systems for mosquitoes kept in a cage or in individual vials and adjusted starvation parameters. Individual mosquitoes offered a bloodmeal in lids of microcentrifuge tubes had the highest blood feeding rate. For field-derived mosquitoes, starvation, or alternatively provision of a 3% salt solution instead of the sugar solution, also consistently improved blood feeding rate, with a minimal impact on survival. We also assessed whether the dissection buffer needed any adjustment for field derived mosquitoes, using spermatheca integrity as a read-out. We found no difference between mosquito strains or buffer, but observed that spermatheca of mosquitoes drown for 24h can still be dissected. In conclusion, our results may be helpful for establishment of new colonies, including in resource-limited settings, studies on fitness of field-derived mosquitoes and on experiments requiring individual level monitoring.”

And in the author summary:

“In this study, we adjusted methods to manipulate field-derived mosquitoes from Cayenne (French Guiana), using the New Orleans reference strain as a control. To improve blood feeding rate, we tested the impact of several blood feeding systems for mosquitoes kept in a cage or in individual vials and adjusted starvation conditions. Our data indicate that the reference strain blood feeds relatively well and is not much impacted by experimental adjustments, while blood feeding of field-derived mosquitoes is substantially affected by blood container and starvation. We also tested differences in dissection using spermatheca integrity as a readout, and did not find any relevance in modifying dissection buffer. Interestingly, spermatheca can still be dissected 24h after mosquitoes drowning, which may be interesting for colony maintenance without sacrificing any female. Our results may enhance experimental reliability of colony establishment and maintenance, including in resource-limited settings and of studies on the fitness of field-derived mosquitoes, including at the individual level.”

Third, the term should be introduced in full at first mention as Aedes aegypti (Ae. aegypti), after which the abbreviated form (Ae. aegypti) should be used consistently throughout the manuscript. The full name should be reintroduced at the first mention in each major section (e.g., Abstract, Introduction, Results) in line with taxonomic writing conventions.

We mentioned full name of *Aedes aegypti* at first mention in the manuscript and then in the abbreviated form.

Finally, while I appreciate the inclusion of a supporting document that outlines the statistical models used, the manuscript would benefit from sharing the underlying raw data. Specifically, information such as the number of mosquitoes tested, those that fed, and those that died should be made available. Providing these data would improve reproducibility and allow others to verify or reanalyze the findings.

We added a Supplementary Table S1 containing the raw data for each experiment and also in the figure caption.

Reviewer #3: In their work “Bridging laboratory and field research: method adjustments to manipulate
field-derived Aedes aegypti,” authors evaluate differences in blood feeding habits and maintenance of tissue integrity for dissections in different media between recently isolated and and laboratory reference strains of Ae. aegypti. In the first experiment, they measure differences in the proportion of blood fed individuals between two Ae. aegypti strains (F1 progeny from the newly isolated Cayenne field colony and the reference laboratory New Orleans strain) using multiple blood feeding devices and methods. In the second experiment, authors evaluate whether various starvation times before a blood meal can impact survival and blood feeding proportions differently between the strains. In the final experiment, the effects of drowning status and dissection solution on spermathecae integrity are evaluated between strains.

I commend the authors for completing this work to compare laboratory and wild populations, as this type of information is critical for mosquito researchers across the globe. My greatest concern is that the introduction does not accurately set the reader up to understand exactly what experiments the authors complete, or what the relevance of each of their experiments is. Experiments lack cohesion, particularly within the introduction. Specifically, it is not clear why experiments only pertaining to blood feeding and tissue dissection were evaluated, or whether it was hypothesized that these factors would differ more than others would between strains. Additionally, there is no mention of dissection media in the introduction. Why this would be included in experiments which only seem to encompass blood feeding effectiveness (in the introduction) is completely unclear. The authors include some motivation and justification for the spermathecae experiment in the results, and this should be moved to the introduction and expounded upon to more clearly motivate the study. While I understand that the scope of this paper is largely exploratory (not testing specific hypotheses), I feel that it is still important to provide the reader with sound reasoning for selecting particular experiments, and the relevance of the items which were chosen to investigate.

Indeed, this was unclear; however, in response to previous comments, we have now better justified our approach by explaining that the idea was to use different readout to adapt to field strains: one for rearing (i.e., blood meal), one for fitness (i.e., starvation), and one for dissection (i.e., spermathecae). This is now better clarified throughout the manuscript to address this point.

Regarding the experiment considering dissection media/drowning on spermathecae integrity, the authors motivate this experiment by indicating that assessing fertilization can be important. However, why can’t burst spermathecae be used to assess fertilization?

It is common practice to burst spermathecae under a cover slip prior to viewing to release sperm, so it is completely unclear why keeping spermathecase “unbursted” is even of interest. Finally, the authors state “Considering conditions to monitor female fertilization, our data indicate that this parameter can still be assessed after death…” but the authors do not actually collect any fertilization data, so I am confused as to how this statement and others like it are justified.

Thanks a lot, we already answered this (see above in the editorial comments). The goal was not to collect fertilization data but use the dissection of spermathecae as a proxy of dissections between laboratory and field mosquitoes. See in the results:

“Ae. aegypti females are typically fertilized during their first mating and then carry spermatozoa in their spermatheca. When rearing mosquitoes, it can also be useful to quantify fertilization, monitoring the presence of sperm cells in dissected spermatheca. As spermathecae sometimes burst during dissection, we used this type of dissection as a readout to account for tissue integrity along the experiment according to mosquito strain and dissection buffer. Two solutions were compared: PBS and Aedes saline, which was formulated to provide optimal osmotic pressure for mosquito dissections [31]. Assessing fertilization is typically interesting when a colony is struggling, and it is necessary to determine why females are not laying eggs. In such cases, it can be useful to also sample females shortly after death, as live females are sometimes too valuable to be collected. Mosquitoes that fall on the bottom of the cage dry out quickly, but those that drown in water can often be dissected several hours later. Therefore, we also tested whether mosquitoes that had drowned 24h beforehand could still be dissected successfully (Figure 4A).”

I also have some concerns regarding the generalizability and significance of the work. While I agree with the authors that the question of how to better perform work in the lab with recently isolated colonies is important, the study only utilizes one field strain and is presented largely as a methods paper. Novel methods are valuable, but they may not be appropriate for this particular journal or appropriate for presentation as a research article. More importantly, however, only utilizing one strain makes it challenging to determine which of the phenotypes are a result of recent field isolation vs. an idiosyncrasy of the genotype of the Cayenne strain. Whether these data could be used to improve the general process of field strain isolation, therefore, is unclear, and this calls into question the broader significance of the work.

We fully understand your concerns. We used Cayenne mosquitoes because they have been well characterized through numerous studies on vector competence, insecticide resistance, and genomics. While extrapolation to other *Aedes aegypti* populations may be somewhat hypothetical, this population—closely related to South American populations—has caused numerous arbovirus epidemics. Additionally, we collected parental larvae from multiple breeding sites across Cayenne to ensure a more heterogeneous genetic pool.

Furthermore, our results can be useful either on their own or to provide parameters to be tested.

Reasons for the selection of different blood feeding methods are also not entirely clear. Table 1 describes details of each method, however it is unclear why only one method (Hemotek capsules) uses collagen membranes, and why only one individual treatment was done. Without the use of one consistent membrane, I wonder whether results are fully a product of different capsule types, or whether membranes have an influence as well. Were the selected membranes both considered to optimize blood feeding to the same extent in previous studies? If so, I would consider inserting this information to make your selection reasoning clear.

You are correct that some differences may arise not only from the container but also from the membrane, but our study aims to compare methods rather than to define the effect of each parameter in different methods.

We added in the method section a paragraph to clarify our choices:

“These methods were selected for several reasons. Firstly, the Hemotek system, which is a standard method for feeding mosquitoes used in many insectaries worldwide, allows for precise control of blood temperature and is typically used with a collagen membrane. However, it is limited when it comes to conducting multiple feedings simultaneously or working in laboratories with scarce equipment. In comparison, we chose Petri dishes along with Parafilm, as they are accessible and inexpensive in laboratory settings. Microtube lids, very common laboratory consumables, were used to test methods for monitoring individual mosquitoes. Using several caps on large cages allowed for a comparison with the two other methods on cages described above.”

Additionally, given that blood volume, total number of capsules per treatment, and type of capsule all vary, I am concerned that temperature of the blood may play a role in blood feeding effectiveness between treatments. While the Hemotek discs have a set temperature, the initial or final temperature of the blood from each treatment was not disclosed. “Activation” of the hand warmers is mentioned, but not specified- were all hand warmers left to “activate” for a certain amount of time, or were they left to reach a certain temperature before being applied to each treatment?

We now added data about temperature in the methods:

“Except for Hemotek that had its own electronic heating system at 37°C, all other treatments were kept warm with gel hand warmers of sodium acetate that were left on top of the blood-feeding devices after getting activated. The hand warmers activate within seconds, reaching 39 ± 1°C and maintaining this temperature throughout the 30min blood meal. Unlike the metal capsule of the Hemotek system that allows the blood to reach a temperature of 37°C within 3 minutes, when using a plastic Petri dish or a tube lid, the blood reaches a temperature of 36°C within about 10 minutes and 3 minutes respectively.”

Other concerns

Please add line numbers to your manuscript.

OK

It is mentioned that “adult mosquitoes were sugar fed on cotton soaked in 10% sucrose solution from the first day of emergence until 24 hours before the start of the experiment, or at specific timings for starvation experiments.” Was 10% sucrose provided ad libitum, or was it provided regularly?

Mosquitoes were sugar fed ad libitum until our experiment. We clarified this point.

The argument that “Determining causal effects of such environmental factors on life history traits becomes almost impossible…” needs to be substantially softened.

There are myriad ecological studies conducted in the field on the role of environmental factors in mosquito biology. This is not “impossible,” it’s just challenging. And some would argue it’s the best way to conduct experiments and any degree of laboratory adaptation leads to results that are less applicable to field populations. I agree that there is value to conducting laboratory experiments, but to assert that proper study of environmental variables on field populations is impossible is incorrect.

Thanks, we did it and changed a little our sentence.

When examining differences in feeding proportions between blood source in Figure 1B, there are multiple factors which could influence results. Not only is the blood source itself different (mechanical warming vs live animal), but the type of blood differs as well (live rodent vs fetal bovine). With this being the case, I find it difficult to agree that blood feeding proportion differences are due to source alone. If “source” includes blood type, I would suggest making this clear to the reader.

We changed the title of the figure1: “. Effect of blood feeding technique on the blood-feeding rate of New Orleans strain vs field-derived *Ae. aegypti* mosquitoes (Cayenne F1).”

In the case that these blood types are not compositionally different from one another, I would suggest including a statement to make this clear to the reader instead. Clarification is needed to make it explicitly clear as to why different blood is used between figure 1A and figure 1B experiments. In-paper reference: “Females of Cayenne F1 Ae. aegypti reached a predicted blood-feeding mean rate of 0.64 (95% CI: 0.42 0.81) on mice, slightly higher than their rate of 0.53 (95% CI: 0.32-0.73) on Hemotek devices (X2 (1)= 4.33, p= 0.04, Figure 1B).” Method reference: “In parallel, the blood-feeding with Hemotek system was performed in the same condition as described above but instead of human serum, blood was mixed with fetal bovine serum (Sigma-Aldrich) and ATP 3.3% (Sigma-Aldrich).”

The figure 1B was from an earlier experiment, and that is the reason why the protocol was not exactly the same. We nevertheless decided to include it, as it provides some additional information.

Under results section “effects of sugar starvation on blood feeding techniques,” the following was stated: “Starvation increases blood-feeding rates but implies fitness costs to Ae. aegypti. We thus looked for a balance between preventing mortality and maximizing blood-feeding rates.” This is a topic that was not prefaced in the intro, and does not seem to have reasoning. I would suggest moving these sentences to the introduction, and including more information to clarify your reason for performing this experiment

We agree, especially since this trade-off between mosquito mortality and increased feeding rate related to starvation was addressed in the discussion. We have therefore removed this sentence from the Results section.

It is unclear how or why the four pre blood-feeding starvation times were selected. Are these times commonly used in other experiments?

These time periods were chosen both because they represent a standard method and to facilitate conducting blood feeds at two different times in a typical working day. This point has been clarified in the methods:

“Four durations of starvation before blood feeding were tested: 0 h (no sugar starvation), 24 h, 30 h, and 48 h. The different deprivation periods were selected based on the literature (typically 24 h) and to accommodate feeding the mosquitoes at convenient timings in the morning and the afternoon.”


Minor concerns

In the abstract the sentence starting “To monitor fertilization…” should be re-worded for syntax and to remove reference to fertilization, as that was not directly assessed at any point.

Thanks, we changed this point.

The sentence beginning “by studying how environmental factors such as temperature…” needs to be re-worded for clarity and syntax.

We changed this sentence in the introduction:

“Studying the ecology and biology of Ae. aegypti is essential to understand the key factors that contribute to its success as a vector and thus to enhance development of targeted and effective vector control strategies preventing arboviral transmission. Notably, environmental factors including temperature, humidity, light exposure and nutrient availability may influence key ecological and biological traits such as mating dynamics, population growth, and fecundity [8]. Determining causal effects of such environmental factors on life history traits is however extremely complex with wild populations in field conditions due to natural variability in environmental variables and between individuals – linked to diet, age, genetics, and body size [9]. Therefore, conducting experiments in controlled laboratory conditions is useful to establish the relative importance of environmental variables on life history traits.”

Please include a description of “the three R’s” or the relevance of this. “artificial feeding systems are increasingly used to follow the principles of Replacement, Reduction, and Refinement (the 3Rs)”

We add a sentence to explain what are the three R’s :

“While most of the earlier studies on mosquito blood feeding were based on the use of live animal hosts, artificial feeding systems are increasingly used to follow the principles of Replacement, Reduction, and Refinement (the 3Rs). They aim to find alternatives to animal testing, to optimize the amount of information obtained from fewer animals, and to adopt methods that alleviate distress respectively [13].”

“beef blood” and “bovine blood” are used interchangeably (see fig. 1 legend and methods). Consider using one term for consistency and clarity.

Done

For your experiment examining the effects of starvation, please clarify whether individuals were provided access to a water source to imbibe before blood meals were provided. In-paper reference: “We starved mosquitoes 0 to 48h before offering them blood in Petri dishes.”

Thanks for this point. In the blood-feeding experiment that we presented, the “sugar starvation” corresponded to a total deprivation water and sugar). We decided to add a new experiment with different types of feeding prior to bloodmeal (sugar 10%, water only, total deprivation, and also a salt solution 3%).

That was explained in the results in the paragraph “Effect of starvation on blood-feeding rates” :

First experiment corresponding to figure 2: “We starved mosquitoes 0 to 48h to sugar and water before offering them blood in Petri dishes.”..

Second experiment corresponding to figure 3: “ We further tested whether hunger or thirst was the essential driver to blood feeding, providing mosquitoes with water only or with a hyperosmotic aqueous solution containing 3% NaCl, keeping 10% sugar solution and total deprivation as non-starved and fully starved controls. In this experiment, starved and non-starved mosquitoes were proposed a blood-meal at the same time to account for potential differences due to circadian-rhythm related issues. We selected relevant conditions from the previous experiment, focusing on Cayenne F1 mosquitoes and on 24h and 30h timings.”

Your statement “Considering the low blood feeding rate of Cayenne F1, we decided to test whether the type of blood meal offered could influence blood feeding success” is unclear. How does low blood feeding proportion in one strain vs. the other lead you to investigate blood meal type? There is an explanatory gap here (my assumption is that you are evaluating whether different blood meal types can improve blood fed individual proportions) .

Thanks, to clarify, that we added to our sentence some details:

“Considering the low blood feeding rate of Cayenne F1, we decided to test whether the type of blood meal offered could influence blood feeding success by evaluating whether different blood meal types could improve blood fed individual proportions.”

In the discussion, the sentence starting “Nevertheless, Ae. aegypti from the field…” does not follow from the previous sentence.

We agree, the word “nevertheless” was deleted.

Discussion reference: “a good balance between mosquito survival and blood feeding rate is obtain after.” Change “obtain” to past tense.

Done

The term “blood feeding rate” can imply that you are measuring the speed at which individuals blood feed at a particular point in time. However, I understand that you actually measure the proportion of blood fed individuals at each time point. This phrasing may be unclear for readers and should be corrected, or defined.

We defined blood feeding rate in the introduction:

“Sugar starvation has been described as a key factor to increase blood feeding rate (proportion of blood fed individuals), the results vary depending on the species, strain, and, most notably, the duration of the sugar starvation period [24].”

The terms “survival” and “survival rate” are used interchangeably. Consider sticking with one term for consistency and ease of understanding for readers.

We corrected that and we only use “survival”.

An n number describing total number of individuals per experiment should be included in figure legends and/or your results section for ease of understanding. Number of replicates should also be included.

We added in figure legends the number of individuals and the number of replicates. For more information, the supplementary table S1 was also added.

It is not explicitly clear in table 1, if one of these feeding regimens was one that was used in the rest of your experiments. Consider including this information in your table 1 description.

In the legend of the table, we added:

“The condition shown in bold was used for the starvation experiment.”

The phrasing “We tested the association between mosquito strain (New Orleans vs Cayenne F1) and the buffer solution used during dissection” seems odd. You are testing success or effectiveness of different buffers in preventing spermathecae from bursting during dissections. I suggest editing the second half of this sentence to clarify.

We agree with that and changed the paragraph as follows:

“As spermathecae sometimes burst during dissection, we used this type of dissection as a readout to account for tissue integrity along the experiment according to mosquito strain and dissection buffer. Two solutions were compared: PBS and Aedes saline, which was formulated to provide optimal osmotic pressure for mosquito dissections [31]. Assessing fertilization is typically interesting when a colony is struggling, and it is necessary to determine why females are not laying eggs. In such cases, it can be useful to also sample females shortly after death, as live females are sometimes too valuable to be collected.”

“Two solutions were compared: “phosphate buffered saline -PBS- and “Aedes saline”, which was previously formulated to provide optimal osmotic pressure for mosquito dissections [24].” Abbreviated terms should be capitalized. The comma after “Aedes saline” should be moved inside of the quotation marks. “previously” can be removed from this sentence.

Done

The Y axis label “expected proportion of intact spermathecae per mosquito” in figure 3 is confusing. Individuals are not plotted in this graph- it depicts means. Also, does the graph show how many spermathecae per individual mosquito were burst (0, 1, 2, or 3), or does it show the number of individuals with burst vs. intact spermathecae? “Expected” is a hypothetical term implying that the experiment had not yet been done (However these data are post-experiment), and should therefore be removed. Consider shortening to “proportion of intact spermathecae.”

We changed the legend onto “Proportion of females with intact spermathecae”

Figure 3 should include methods that describe whether the same person performed all dissections. Everyone performs dissections differently, and various experimenters would add a complex variable (human error and/or habits) and weaken the validity of these results.

In your figure 3 methods, you describe that “New Orleans females were collected from cages where they had spent between 10 and 20 days with males, while field-collected females were not necessarily given time to mate in the laboratory.” I do not understand why these individuals were left to mate with males for such a long period of time (especially compared to all other experiments), or why field collected individuals were not given time to mate. Were these individuals those directly collected from the field, or were they F1 progeny of field populations as in your other experiments? As it is currently written, I understand it to be the former, which would introduce variables such as age and environmental conditions. If it is the latter, it should be explained what “necessarily” means, and why the experiment was done this way.

We did the whole spermatheca experiment again and hope that the new explanations will convince the reviewer. A single person has performed the second set of experiments that was kept in the revised manuscript.

“Establishment of experimental conditions for spermatheca dissection.” In your figure 3 legend is odd phrasing. Direct language that explicitly states what the reader is looking at will mitigate confusion. Consider changing this to “Effects of drowning status and dissection solution on spermathecae integrity.”

Thank you, we followed your comment. The title of the figure 4 (ex-figure 3) is now:

“Effects of drowning status and dissection solution on spermathecae integrity.”

The image of the individual 1.5 microtube feeder should be more zoomed in so the viewer can see precisely how it was set up.

Done
