# Supplementary material for: Bridging laboratory and field research: Method adjustments to blood feed field-derived Aedes aegypti
Source: PLoS Negl Trop Dis. 2026 May 18;20(5):e0013339. doi: 10.1371/journal.pntd.0013339 (PMC13249168; doi:10.1371/journal.pntd.0013339)
Supplement: Attachment — Submitted filename: Response_to_reviewers_auresp_2.docx [file pntd.0013339.s008.docx]

**Additional Editor Comments :**

Thank you for submitting your revised manuscript to PLOS Neglected Tropical Diseases. We appreciate the effort you have put into addressing the concerns raised in the previous round of review. Your manuscript has been evaluated by three reviewers, two of whom are satisfied with the revisions and recommend acceptance. However, one reviewer has raised concerns that we believe must be addressed before the manuscript can be accepted for publication. We therefore invite you to submit a major revision.

The reviewer's comments are included in full below, however here are the key points that will require attention:
Isotonic solutions experiment: The reviewer finds the motivation for this experiment to be underdeveloped. The manuscript does not cite relevant literature, does not justify using spermatheca integrity as a readout for tissue integrity more broadly, and does not explain why tissue integrity is relevant to the study's aims. More critically, the conclusion that fertilization status can be assessed post-mortem is not supported by the data, as fertilization itself was never measured. The reviewer recommends removing this experiment from the manuscript. We agree that this is worth serious consideration; if the authors wish to retain it, a substantially stronger justification is required.

We now removed this experiment, as well as the related text and references. Consequently we changed the title of the paper into “Bridging laboratory and field research: method adjustments to blood feed field-derived *Aedes aegypti*”.

Wording error (lines 127–129): The text incorrectly describes the independent variable in the blood feeding experiment as "type of blood meal" when all meals are human-derived, and the variable is, in fact, the method of blood feeding. This should be corrected.

We totally agree and changed our sentence in the text in the first section of results.

Clarity of experimental design for Figure 1A: It must be made unambiguous whether the Petri dish experiments were performed as part of the same experiment as the Hemotek and lid conditions, or separately. The presentation (either graphically or in the text) should be updated accordingly.
As you rightly pointed out, this section was unclear due to an early discussion of the Petri dish. Since all blood feeding methods were performed simultaneously, we have restructured this part for greater clarity. See in the text :

“We first quantified the difference in blood-feeding rate between Ae. aegypti mosquitoes of the New Orleans reference strain and the progeny of Ae. aegypti mosquitoes collected as larvae, referred to here as “Cayenne F1”. We compared several blood-feeding methods: we offered blood in a Hemotek capsule, in a Petri dish, in several lids of Eppendorf tubes laid on the cage (‘Collective lids’) or in one lid offered to a mosquito kept in an individual tube (‘Individual lids’, Table 1). These containers were placed upside down on the cages so that mosquitoes could bite through an artificial membrane (Table 1). The analysis revealed a significant effect of the interaction between mosquito strains and the blood-feeding technique on the blood-feeding success (X2(3)=11.26, p=0.01, Figure 1A, Table A in S1 Text), which indicates that the efficiency of blood-feeding methods differed depending on mosquito origin. Specifically, this model predicted a mean feeding rate of 0.73 (95% CI: 0.63-0.81) for the New Orleans strain, and of 0.32 (95% CI: 0.23-0.43) for Cayenne F1. When we offered for instance blood in Petri dish, we observed a strong effect of the mosquito strain, with a mean feeding rate of 0.7 for New Orleans and only 0.21 for Cayenne F1 (Figure 1A; β=-2.07, Z=-5.11, p<0.01; see table A in S1 Text for fitted values). Regarding the blood-feeding method, most Post-Hoc pairwise comparisons did not differ significantly from one another within the New Orleans strain (Figure 1; Table B in S1 Text). However, Post-Hoc pairwise comparisons of blood-feeding methods within the Cayenne F1 strain showed that “individual lids” resulted in the highest blood-feeding rate (0.59, 95% CI: 0.45-0.71; Figure 1A; p<0.001; Table B in S1 Text), compared to the other methods such as collective lids (0.30, 95% CI: 0.20-0.42), Hemotek (0.24, 95% CI: 0.15-0.36), and Petri dish (0.21, 95% CI: 0.13-0.32) .”

Lines 165–167: The authors should revisit this section, as the statement about strain influencing starvation time is logically inconsistent, given that starvation time is an independent variable. Please clarify the intended meaning.

We have revised the text to eliminate this confusion:

“Such was the effect of the *Ae. aegypti* strain (X2(1)=4.19, p=0.04; Figure 2A), and of the pre-blood-feeding starvation time (X2(3)=111.71, p<0.001; Figure 2A) on survival rate.”

Figure 2 legend: The legend should be expanded to address the significant difference between strains and to describe the primary findings of panel 2B, so that the figure is self-contained.

Instead of expanding the legend, we have included a statistical summary table in figure 2, reporting first the treatment × strain interactions and then the post-hoc pairwise comparisons.

**Reviewer comments**

Use this section to provide overall comments, discuss strengths/weaknesses of the study, novelty, significance, general execution and scholarship. You may also include additional comments for the author, including concerns about dual publication, research ethics, or publication ethics. If requesting major revision, please articulate the new experiments that are needed.

Reviewer #1: This is a marked improvement from the original submission, and I am very pleased to have gotten the chance to see it in its revised form. The authors should be commended for the work that they have done to put this revised version together. I believe it meets the standards for publication as-is. It could, if one were so inclined, benefit from some light copy editing to standardize some of the English (e.g. swapping 'drowned' for 'drown'). However, the text is clear and readable to a native speaker as-is.

We sincerely appreciate your positive feedback and are grateful for the time and care you’ve taken to review our revised manuscript.

The term 'drowned' no longer appears anywhere in the manuscript, as we removed the spermatheca experiment.

Reviewer #2: The authors addressed all the concerns I had

Thanks.

Reviewer #3: I appreciate the improvements the authors have made to the manuscript. I still have some concerns that need to be addressed, however.

There is still insufficient motivation presented for studying the question of whether different isotonic solutions produce different results during spermathecae dissection. The authors state that different isotonic solutions have been used for this purpose, but they do not motivate the study in any way. Is it a relevant question to query whether one solution is better than the other? Is there any literature that suggests that one may be preferable to the other? Is there a reason to think that different solutions could potentially differ in their effects on recently isolated strains? Moreover, the authors indicate that they used spermatheca integrity as a readout for the effects of these solutions on mosquito tissue overall. But what prior research suggests that spermatheca integrity an appropriate readout for overall tissue integrity? Also, perhaps even more important, why is tissue integrity a relevant question in this study? Finally, the authors conclude from these data that female fertilization can be assessed after death given that they dissected spermathecae from drowned females. However, they never actually assessed fertilization, so this statement is not well supported. I strongly suggest the authors simply remove this experiment from the study. It does not appear to offer much benefit and instead introduces substantial confusion.

We now removed this experiment, as well as the related text and references. Consequently we changed the title of the paper into “Bridging laboratory and field research: method adjustments to blood feed field-derived *Aedes aegypti*”.

On line 127-129, the authors indicate that they are testing whether the type of blood meal offered could influence blood feeding success. However, in the next section, they do not evaluate type of blood meal but rather method of blood feeding (since all blood meals in figure 1A are human derived). The wording here should be changed to reflect this.

We totally agree and changed our sentence in the text in the first section of results.

It is not clear to me whether the Petri dish experiments in figure 1A were performed separately from the other three feeding methods. Based on the way the results are written, the reader is led to believe the Petri dish experiments were performed first and then the Hemotek and lids experiments were performed together in a subsequent experiment. If the Petri dish experiment was performed separately from the other methods, it should be in its own graph in Fig. 1. If all feeding methods presented in Fig. 1A were performed as part of the same experiment, lines 120-143 should be rewritten to make this clear.

As you rightly pointed out, this section was unclear due to an early discussion of the Petri dish. Since all blood feeding methods were performed simultaneously, we have restructured this part for greater clarity. See in the text :

“We first quantified the difference in blood-feeding rate between Ae. aegypti mosquitoes of the New Orleans reference strain and the progeny of Ae. aegypti mosquitoes collected as larvae, referred to here as “Cayenne F1”. We compared several blood-feeding methods: we offered blood in a Hemotek capsule, in a Petri dish, in several lids of Eppendorf tubes laid on the cage (‘Collective lids’) or in one lid offered to a mosquito kept in an individual tube (‘Individual lids’, Table 1). These containers were placed upside down on the cages so that mosquitoes could bite through an artificial membrane (Table 1). The analysis revealed a significant effect of the interaction between mosquito strains and the blood-feeding technique on the blood-feeding success (X2(3)=11.26, p=0.01, Figure 1A, Table A in S1 Text), which indicates that the efficiency of blood-feeding methods differed depending on mosquito origin. Specifically, this model predicted a mean feeding rate of 0.73 (95% CI: 0.63-0.81) for the New Orleans strain, and of 0.32 (95% CI: 0.23-0.43) for Cayenne F1. When we offered for instance blood in Petri dish, we observed a strong effect of the mosquito strain, with a mean feeding rate of 0.7 for New Orleans and only 0.21 for Cayenne F1 (Figure 1A; β=-2.07, Z=-5.11, p<0.01; see table A in S1 Text for fitted values). Regarding the blood-feeding method, most Post-Hoc pairwise comparisons did not differ significantly from one another within the New Orleans strain (Figure 1; Table B in S1 Text). However, Post-Hoc pairwise comparisons of blood-feeding methods within the Cayenne F1 strain showed that “individual lids” resulted in the highest blood-feeding rate (0.59, 95% CI: 0.45-0.71; Figure 1A; p<0.001; Table B in S1 Text), compared to the other methods such as collective lids (0.30, 95% CI: 0.20-0.42), Hemotek (0.24, 95% CI: 0.15-0.36), and Petri dish (0.21, 95% CI: 0.13-0.32) .”

In line 165-167, I do not understand what the authors are trying to say. Figure 2A does not appear to present data on blood feeding rate, and how could strain affect blood feeding starvation time when starvation time is an independent variable? The authors need to revisit this sentence and reevaluate what they were trying to say.

We have revised the text to eliminate this confusion:

“Such was the effect of the *Ae. aegypti* strain (X2(1)=4.19, p=0.04; Figure 2A), and of the pre-blood-feeding starvation time (X2(3)=111.71, p<0.001; Figure 2A) on survival rate.”

In the legend for figure 2, the authors give details as to significant differences and survival rate over time, but do not speak to the significant difference between strain. They also give no detail about the results of 2B. Figure legends should stand alone, therefore, some discussion of the primary findings of figure 2B is important to include in the figure legend.

Instead of expanding the legend, we have included a statistical summary table in figure 2, reporting first the treatment × strain interactions and then the post-hoc pairwise comparisons.
