# Supplementary material for: Bridging laboratory and field research: Method adjustments to blood feed field-derived Aedes aegypti
Source: PLoS Negl Trop Dis. 2026 May 18;20(5):e0013339. doi: 10.1371/journal.pntd.0013339 (PMC13249168; doi:10.1371/journal.pntd.0013339)
Supplement: Peer review history

# Peer review: Decision Letter 0

**DOI:** 10.1371/journal.pntd.0013339.r001

**Authors:** Paul Mireji

11 Sep 2025

-->PNTD-D-25-01115

Bridging laboratory and field research: method adjustments to manipulate field-derived *Aedes aegypti*

PLOS Neglected Tropical Diseases

Dear Dr. Gendrin,

Thank you for submitting your manuscript to PLOS Neglected Tropical Diseases. After careful consideration, we feel that it has merit but does not fully meet PLOS Neglected Tropical Diseases's publication criteria as it currently stands. Therefore, we invite you to submit a revised version of the manuscript that addresses the points raised during the review process.

Please submit your revised manuscript within 60 days Nov 10 2025 11:59PM. If you will need more time than this to complete your revisions, please reply to this message or contact the journal office at plosntds@plos.org. When you're ready to submit your revision, log on to https://www.editorialmanager.com/pntd/ and select the 'Submissions Needing Revision' folder to locate your manuscript file.

Please include the following items when submitting your revised manuscript:

* A rebuttal letter that responds to each point raised by the editor and reviewer(s). You should upload this letter as a separate file labeled 'Response to Reviewers'. This file does not need to include responses to any formatting updates and technical items listed in the 'Journal Requirements' section below.

* A marked-up copy of your manuscript that highlights changes made to the original version. You should upload this as a separate file labeled 'Revised Manuscript with Track Changes'.

* An unmarked version of your revised paper without tracked changes. You should upload this as a separate file labeled 'Manuscript'.

If you would like to make changes to your financial disclosure, competing interests statement, or data availability statement, please make these updates within the submission form at the time of resubmission. Guidelines for resubmitting your figure files are available below the reviewer comments at the end of this letter.

We look forward to receiving your revised manuscript.

Kind regards,

Geoffrey M. Attardo

Academic Editor

PLOS Neglected Tropical Diseases

Paul Mireji

Section Editor

PLOS Neglected Tropical Diseases

Shaden Kamhawi

co-Editor-in-Chief

PLOS Neglected Tropical Diseases

orcid.org/0000-0003-4304-636XX

Paul Brindley

co-Editor-in-Chief

PLOS Neglected Tropical Diseases

orcid.org/0000-0003-1765-0002

**Additional Editor Comments:**

Dear Dr. Gendrin,

Thank you for your submission to PLoS NTDs and my apologies for the delay in getting feedback on your manuscript to you. The reviewers all felt that your manuscript presenting studies on work with field versus lab mosquitoes is valuable, relevant and overall well-executed. However, there are aspects of the manuscript that require revision to improve clarity/cohesion, justification of experimental design decisions, and thoroughness in reporting on methods and sample sizes. No additional experimental work is requested, but the reviewers recommend revisions to clarify these aspects.

Specifically, the reviewers would like you to address the following:

The hypotheses behind the different aspects of the study are present, but not always clearly articulated and need to be more explicitly stated

The introduction does not set up the rationale or relationship between the experiments. For example, why were spermathecae integrity and dissection media were tested along with feeding assays? The relevance/significance of the experiments should be clarified in the introduction rather than the results.

Additional clarity on the materials and methods is needed. Specifically:

Samples sizes on the spermathecae experiments need to be reported.

Description of the rationale behind the use of different feeding methodologies.

Clarification of the rationale behind starvation times.

Details on experiments need to be included including blood temperatures and timing on activation of hand warmers.

Clarification on if the mosquitoes were F1 progeny or directly from field collections.

Presentation of the results could be improved:

Sample sizes and the number of replicates should be included.

The Y-axis in figure 3 "expected proportion of intact spermathecae" is unclear and should be revised for clarity

Blood source comparisions in figure 1 should be clarified.

There is an inconsistency between the feeding time listed in Figure 1 (45 mins) versus that described in the materials and methods (30 mins)

Please include the raw data such as the number of mosquitoes tested, fed, observed mortality, for reproducibility purposes.

Over statement/interpretation of conclusions:

Discussion on the limitations of using a single field strain and consideration of variability between genetically distinct strains would be helpful.

Statements about environmental factors being "impossible" to study in the field should be softened.

Clarification of why unburst spermathecae are of interest when microscopic examination of burst spermathecae is standard for fertiliy status assessment.

More discussion on the differences between colony and near-field mosquitoes in situations such as sugar starvation.

Expanded discussion on the broader implications for field and lab practices given your findings.

There were also a number of stylistic revisions recommended:

Make sure to include the main findings and explicitly state the studies goals in the abstract and author summary.

Introduce Aedes aegypti in full at its first mention and abbreviate later mentions.

Make sure terminology is consistent. For example: "beef blood" versus "bovine blood"; "survival" versus "survival rate".

Clarify phrasing in certain places as described by the reviewers.

Thank you again for your submission and we look forward to receiving the revised version of your manuscript.

Best wishes,

Geoff Attardo

**Journal Requirements:**

1) We do not publish any copyright or trademark symbols that usually accompany proprietary names, eg ©,  ®, or TM  (e.g. next to drug or reagent names). Therefore please remove all instances of trademark/copyright symbols throughout the text, including:

- © on page: 10.

- ® on pages: 3, 9, 10, and 12.

2) Please upload all main figures as separate Figure files in .tif or .eps format. For more information about how to convert and format your figure files please see our guidelines:

https://journals.plos.org/plosntds/s/figures

3) Some material included in your submission may be copyrighted. According to PLOSu2019s copyright policy, authors who use figures or other material (e.g., graphics, clipart, maps) from another author or copyright holder must demonstrate or obtain permission to publish this material under the Creative Commons Attribution 4.0 International (CC BY 4.0) License used by PLOS journals. Please closely review the details of PLOSu2019s copyright requirements here: PLOS Licenses and Copyright. If you need to request permissions from a copyright holder, you may use PLOS's Copyright Content Permission form.

Please respond directly to this email and provide any known details concerning your material's license terms and permissions required for reuse, even if you have not yet obtained copyright permissions or are unsure of your material's copyright compatibility. Once you have responded and addressed all other outstanding technical requirements, you may resubmit your manuscript within Editorial Manager.

Potential Copyright Issues:

- Please confirm (a) that you are the photographer of Figure 4., or (b) provide written permission from the photographer to publish the photo(s) under our CC BY 4.0 license.

**Reviewers' Comments:**

Reviewer's Responses to Questions

**Key Review Criteria Required for Acceptance?**

As you describe the new analyses required for acceptance, please consider the following:

**Methods:**

-Are the objectives of the study clearly articulated with a clear testable hypothesis stated?

-Is the study design appropriate to address the stated objectives?

-Is the population clearly described and appropriate for the hypothesis being tested?

-Is the sample size sufficient to ensure adequate power to address the hypothesis being tested?

-Were correct statistical analysis used to support conclusions?

-Are there concerns about ethical or regulatory requirements being met?

Reviewer #1: - The hypotheses are present, though not always clearly stated.

- The study design, overall, appears appropriate

- The populations are clearly defined. It would be ideal to have an established, long-term colony of local mosquitoes as control, but the New Orleans strain is likely a good proxy as a regional, relatively recently derived strain

- It is unclear if the sample size is sufficient. Sample size for the spermathecae experiments does not appear to be reported. Thus, the difference found, though significant, is unclear if it should be dismissed as an artifact of low sample size or if it's a true difference.

- Suitable statistical tests were used, though it would have also have been suitable to perform non model-based statistics with the appropriate classical statistical checks. This might improve interpretation by a broader audience. It was nice to see the experimental conditions included as random effects, though, as an additional check.

- No ethical or regulatory concerns were identified.

Reviewer #2: yes

Reviewer #3: (No Response)

**********

**Results**

-Does the analysis presented match the analysis plan?

-Are the results clearly and completely presented?

-Are the figures (Tables, Images) of sufficient quality for clarity?

Reviewer #1: - The analysis matches the analysis plan

- The results are completely presented, however clarity is limited by words and turns of phrase covered in the Methods section that is not until later.

- Figure 1 states that mosquitoes were fed for 45 minutes, while the Methods section states 30 minutes.

Reviewer #2: yes

Reviewer #3: (No Response)

**********

**Conclusions:**

-Are the conclusions supported by the data presented?

-Are the limitations of analysis clearly described?

-Do the authors discuss how these data can be helpful to advance our understanding of the topic under study?

-Is public health relevance addressed?

Reviewer #1: - The conclusions are supported by the data presented (with the caveat of the missing sample size previously mentioned).

- Limitations not discussed include not using a colony of locally-derived Ae. aegypti (though maybe not practical for this study, others have show significant behavioral and genetic differences in populations of this species within a single country over relatively short regional distances); the potential that the buffer/spermatheca issue could be a small sample size or sampling issue (i.e. no physiological or other basis for this difference is hypothesized, so it reads like an experimental artifact that seemed odd, so it made it into the paper); the limitation of different membranes for different feeders is not discussed (we use Parafilm membranes on Hemoteks without issue, so it is unclear as to why this extra variability was added); and a discussion point about near-field vs. colony mosquitoes and sugar starvation is missing (i.e. colony mosquitoes are reared with ad libitum access to sucrose while near-field mosquitoes are not used to having that access. There are likely physiological differences associated with this that help explain why the colony mosquitoes cannot tolerate extended sugar starvation in the way that the F1 colonies can. While the timing of the meals are a common critique, in our hands this species readily feeds in the field and in the lab at all hours at relatively consistent levels. So, I would encourage the authors to think more about why that difference may have shown up as it is interesting).

- Public health relevance is addressed in the Discussion, though more could be said.

Reviewer #2: yes

Reviewer #3: (No Response)

**********

**Editorial and Data Presentation Modifications?**

Use this section for editorial suggestions as well as relatively minor modifications of existing data that would enhance clarity. If the only modifications needed are minor and/or editorial, you may wish to recommend “Minor Revision” or “Accept”.

Reviewer #1: The order of the manuscript sections could be revised for clarity and minor grammatical items throughout.

Reviewer #2: Minor revision

Reviewer #3: (No Response)

**********

**Summary and General Comments:**

Use this section to provide overall comments, discuss strengths/weaknesses of the study, novelty, significance, general execution and scholarship. You may also include additional comments for the author, including concerns about dual publication, research ethics, or publication ethics. If requesting major revision, please articulate the new experiments that are needed.

Reviewer #1: The authors put forth several connected studies that investigate the differences between their local F1 Ae. aegypti and a standardized colony strain. Consistent production of quality, high number near-field material is important to the field, and any tools and protocols that can help further that work are certainly welcome.

The authors have clearly put forth a great deal of effort into compiling these studies and this manuscript and should be commended. I believe the previous comments cover the major points of concern. If there are justifications for the use of different membranes and the sample size for the spermatheca dissections was sufficient and seems to be a true difference with some plausible explanation, then I do not believe that any experiments need to be repeated to major revisions undertaken.

I believe the strength of this manuscript lies in the feeding success rates, sugar starvation times, and feeding devices. A manuscript with more detail on those points demonstrating a reproducible and reliable method for bringing in and generating F1s would be very helpful to the field.

Reviewer #2: Overall, this is a well-written manuscript that addresses an important research problem involving mosquito studies in low-cost settings. The study is relevant and will provide valuable insights for future work on vector competence and related laboratory and field studies.

That said, I identified several areas that would benefit from revision. First, the abstract and author summary are incomplete, as they do not include the main findings of the study. Both sections currently introduce the topic and highlight its importance but fail to communicate the results, which are essential for readers to quickly grasp the contribution and impact of the work.

Second, the main goal of the study is not clearly and explicitly stated in either the abstract or the author summary. While it may be implied, it should be presented in straightforward terms so that readers immediately understand the central objective of the work.

Third, the term should be introduced in full at first mention as Aedes aegypti (Ae. aegypti), after which the abbreviated form (Ae. aegypti) should be used consistently throughout the manuscript. The full name should be reintroduced at the first mention in each major section (e.g., Abstract, Introduction, Results) in line with taxonomic writing conventions.

Finally, while I appreciate the inclusion of a supporting document that outlines the statistical models used, the manuscript would benefit from sharing the underlying raw data. Specifically, information such as the number of mosquitoes tested, those that fed, and those that died should be made available. Providing these data would improve reproducibility and allow others to verify or reanalyze the findings.

Reviewer #3: In their work “Bridging laboratory and field research: method adjustments to manipulate

field-derived Aedes aegypti,” authors evaluate differences in blood feeding habits and maintenance of tissue integrity for dissections in different media between recently isolated and and laboratory reference strains of Ae. aegypti. In the first experiment, they measure differences in the proportion of blood fed individuals between two Ae. aegypti strains (F1 progeny from the newly isolated Cayenne field colony and the reference laboratory New Orleans strain) using multiple blood feeding devices and methods. In the second experiment, authors evaluate whether various starvation times before a blood meal can impact survival and blood feeding proportions differently between the strains. In the final experiment, the effects of drowning status and dissection solution on spermathecae integrity are evaluated between strains.

I commend the authors for completing this work to compare laboratory and wild populations, as this type of information is critical for mosquito researchers across the globe. My greatest concern is that the introduction does not accurately set the reader up to understand exactly what experiments the authors complete, or what the relevance of each of their experiments is. Experiments lack cohesion, particularly within the introduction. Specifically, it is not clear why experiments only pertaining to blood feeding and tissue dissection were evaluated, or whether it was hypothesized that these factors would differ more than others would between strains. Additionally, there is no mention of dissection media in the introduction. Why this would be included in experiments which only seem to encompass blood feeding effectiveness (in the introduction) is completely unclear. The authors include some motivation and justification for the spermathecae experiment in the results, and this should be moved to the introduction and expounded upon to more clearly motivate the study. While I understand that the scope of this paper is largely exploratory (not testing specific hypotheses), I feel that it is still important to provide the reader with sound reasoning for selecting particular experiments, and the relevance of the items which were chosen to investigate.

Regarding the experiment considering dissection media/drowning on spermathecae integrity, the authors motivate this experiment by indicating that assessing fertilization can be important. However, why can’t burst spermathecae be used to assess fertilization? It is common practice to burst spermathecae under a cover slip prior to viewing to release sperm, so it is completely unclear why keeping spermathecase “unbursted” is even of interest. Finally, the authors state “Considering conditions to monitor female fertilization, our data indicate that this parameter can still be assessed after death…” but the authors do not actually collect any fertilization data, so I am confused as to how this statement and others like it are justified.

I also have some concerns regarding the generalizability and significance of the work. While I agree with the authors that the question of how to better perform work in the lab with recently isolated colonies is important, the study only utilizes one field strain and is presented largely as a methods paper. Novel methods are valuable, but they may not be appropriate for this particular journal or appropriate for presentation as a research article. More importantly, however, only utilizing one strain makes it challenging to determine which of the phenotypes are a result of recent field isolation vs. an idiosyncrasy of the genotype of the Cayenne strain. Whether these data could be used to improve the general process of field strain isolation, therefore, is unclear, and this calls into question the broader significance of the work.

Reasons for the selection of different blood feeding methods are also not entirely clear. Table 1 describes details of each method, however it is unclear why only one method (Hemotek capsules) uses collagen membranes, and why only one individual treatment was done. Without the use of one consistent membrane, I wonder whether results are fully a product of different capsule types, or whether membranes have an influence as well. Were the selected membranes both considered to optimize blood feeding to the same extent in previous studies? If so, I would consider inserting this information to make your selection reasoning clear. Additionally, given that blood volume, total number of capsules per treatment, and type of capsule all vary, I am concerned that temperature of the blood may play a role in blood feeding effectiveness between treatments. While the Hemotek discs have a set temperature, the initial or final temperature of the blood from each treatment was not disclosed. “Activation” of the hand warmers is mentioned, but not specified- were all hand warmers left to “activate” for a certain amount of time, or were they left to reach a certain temperature before being applied to each treatment?

Other concerns

Please add line numbers to your manuscript.

It is mentioned that “adult mosquitoes were sugar fed on cotton soaked in 10% sucrose solution from the first day of emergence until 24 hours before the start of the experiment, or at specific timings for starvation experiments.” Was 10% sucrose provided ad libitum, or was it provided regularly?

The argument that “Determining causal effects of such environmental factors on life history traits becomes almost impossible…” needs to be substantially softened. There are myriad ecological studies conducted in the field on the role of environmental factors in mosquito biology. This is not “impossible,” it’s just challenging. And some would argue it’s the best way to conduct experiments and any degree of laboratory adaptation leads to results that are less applicable to field populations. I agree that there is value to conducting laboratory experiments, but to assert that proper study of environmental variables on field populations is impossible is incorrect.

When examining differences in feeding proportions between blood source in Figure 1B, there are multiple factors which could influence results. Not only is the blood source itself different (mechanical warming vs live animal), but the type of blood differs as well (live rodent vs fetal bovine). With this being the case, I find it difficult to agree that blood feeding proportion differences are due to source alone. If “source” includes blood type, I would suggest making this clear to the reader. In the case that these blood types are not compositionally different from one another, I would suggest including a statement to make this clear to the reader instead. Clarification is needed to make it explicitly clear as to why different blood is used between figure 1A and figure 1B experiments. In-paper reference: “Females of Cayenne F1 Ae. aegypti reached a predicted blood-feeding mean rate of 0.64 (95% CI: 0.42 0.81) on mice, slightly higher than their rate of 0.53 (95% CI: 0.32-0.73) on Hemotek devices (X2 (1)= 4.33, p= 0.04, Figure 1B).” Method reference: “In parallel, the blood-feeding with Hemotek system was performed in the same condition as described above but instead of human serum, blood was mixed with fetal bovine serum (Sigma-Aldrich) and ATP 3.3% (Sigma-Aldrich).”

Under results section “effects of sugar starvation on blood feeding techniques,” the following was stated: “Starvation increases blood-feeding rates but implies fitness costs to Ae. aegypti. We thus looked for a balance between preventing mortality and maximizing blood-feeding rates.” This is a topic that was not prefaced in the intro, and does not seem to have reasoning. I would suggest moving these sentences to the introduction, and including more information to clarify your reason for performing this experiment

It is unclear how or why the four pre blood-feeding starvation times were selected. Are these times commonly used in other experiments?

Minor concerns

In the abstract the sentence starting “To monitor fertilization…” should be re-worded for syntax and to remove reference to fertilization, as that was not directly assessed at any point.

The sentence beginning “by studying how environmental factors such as temperature…” needs to be re-worded for clarity and syntax.

Please include a description of “the three R’s” or the relevance of this. “artificial feeding systems are increasingly used to follow the principles of Replacement, Reduction, and Refinement (the 3Rs)”

“beef blood” and “bovine blood” are used interchangeably (see fig. 1 legend and methods). Consider using one term for consistency and clarity.

For your experiment examining the effects of starvation, please clarify whether individuals were provided access to a water source to imbibe before blood meals were provided. In-paper reference: “We starved mosquitoes 0 to 48h before offering them blood in Petri dishes.”

Your statement “Considering the low blood feeding rate of Cayenne F1, we decided to test whether the type of blood meal offered could influence blood feeding success” is unclear. How does low blood feeding proportion in one strain vs. the other lead you to investigate blood meal type? There is an explanatory gap here (my assumption is that you are evaluating whether different blood meal types can improve blood fed individual proportions) .

In the discussion, the sentence starting “Nevertheless, Ae. aegypti from the field…” does not follow from the previous sentence.

Discussion reference: “a good balance between mosquito survival and blood feeding rate is obtain after.” Change “obtain” to past tense.

The term “blood feeding rate” can imply that you are measuring the speed at which individuals blood feed at a particular point in time. However, I understand that you actually measure the proportion of blood fed individuals at each time point. This phrasing may be unclear for readers and should be corrected, or defined.

The terms “survival” and “survival rate” are used interchangeably. Consider sticking with one term for consistency and ease of understanding for readers.

An n number describing total number of individuals per experiment should be included in figure legends and/or your results section for ease of understanding. Number of replicates should also be included.

It is not explicitly clear in table 1, if one of these feeding regimens was one that was used in the rest of your experiments. Consider including this information in your table 1 description.

The phrasing “We tested the association between mosquito strain (New Orleans vs Cayenne F1) and the buffer solution used during dissection” seems odd. You are testing success or effectiveness of different buffers in preventing spermathecae from bursting during dissections. I suggest editing the second half of this sentence to clarify.

“Two solutions were compared: “phosphate buffered saline -PBS- and “Aedes saline”, which was previously formulated to provide optimal osmotic pressure for mosquito dissections [24].” Abbreviated terms should be capitalized. The comma after “Aedes saline” should be moved inside of the quotation marks. “previously” can be removed from this sentence.

The Y axis label “expected proportion of intact spermathecae per mosquito” in figure 3 is confusing. Individuals are not plotted in this graph- it depicts means. Also, does the graph show how many spermathecae per individual mosquito were burst (0, 1, 2, or 3), or does it show the number of individuals with burst vs. intact spermathecae? “Expected” is a hypothetical term implying that the experiment had not yet been done (However these data are post-experiment), and should therefore be removed. Consider shortening to “proportion of intact spermathecae.”

Figure 3 should include methods that describe whether the same person performed all dissections. Everyone performs dissections differently, and various experimenters would add a complex variable (human error and/or habits) and weaken the validity of these results.

In your figure 3 methods, you describe that “New Orleans females were collected from cages where they had spent between 10 and 20 days with males, while field-collected females were not necessarily given time to mate in the laboratory.” I do not understand why these individuals were left to mate with males for such a long period of time (especially compared to all other experiments), or why field collected individuals were not given time to mate. Were these individuals those directly collected from the field, or were they F1 progeny of field populations as in your other experiments? As it is currently written, I understand it to be the former, which would introduce variables such as age and environmental conditions. If it is the latter, it should be explained what “necessarily” means, and why the experiment was done this way.

“Establishment of experimental conditions for spermatheca dissection.” In your figure 3 legend is odd phrasing. Direct language that explicitly states what the reader is looking at will mitigate confusion. Consider changing this to “Effects of drowning status and dissection solution on spermathecae integrity.”

The image of the individual 1.5 microtube feeder should be more zoomed in so the viewer can see precisely how it was set up.

**********

PLOS authors have the option to publish the peer review history of their article (what does this mean?). If published, this will include your full peer review and any attached files.

If you choose “no”, your identity will remain anonymous but your review may still be made public.

**Do you want your identity to be public for this peer review?** For information about this choice, including consent withdrawal, please see our Privacy Policy.

Reviewer #1: No

Reviewer #2: No

Reviewer #3: No

[NOTE: If reviewer comments were submitted as an attachment file, they will be attached to this email and accessible via the submission site. Please log into your account, locate the manuscript record, and check for the action link "View Attachments". If this link does not appear, there are no attachment files.]

**Figure resubmission:**

-->While revising your submission, we strongly recommend that you use PLOS’s NAAS tool (https://ngplosjournals.pagemajik.ai/artanalysis) to test your figure files. NAAS can convert your figure files to the TIFF file type and meet basic requirements (such as print size, resolution), or provide you with a report on issues that do not meet our requirements and that NAAS cannot fix.--> -->

After uploading your figures to PLOS’s NAAS tool - https://ngplosjournals.pagemajik.ai/artanalysis, NAAS will process the files provided and display the results in the "Uploaded Files" section of the page as the processing is complete. If the uploaded figures meet our requirements (or NAAS is able to fix the files to meet our requirements), the figure will be marked as "fixed" above. If NAAS is unable to fix the files, a red "failed" label will appear above. When NAAS has confirmed that the figure files meet our requirements, please download the file via the download option, and include these NAAS processed figure files when submitting your revised manuscript.-->

**Reproducibility:**

To enhance the reproducibility of your results, we recommend that authors of applicable studies deposit laboratory protocols in protocols.io, where a protocol can be assigned its own identifier (DOI) such that it can be cited independently in the future. Additionally, PLOS ONE offers an option to publish peer-reviewed clinical study protocols. Read more information on sharing protocols at https://plos.org/protocols?utm_medium=editorial-email&utm_source=authorletters&utm_campaign=protocols

-->

Attachment Submitted filename: review..docx

# Author comment: Author response to Decision Letter 1

**DOI:** 10.1371/journal.pntd.0013339.r002

30 Jan 2026

Attachment Submitted filename: Response to reviewers.docx

# Peer review: Decision Letter 1

**DOI:** 10.1371/journal.pntd.0013339.r003

**Authors:** Paul Mireji

18 Mar 2026

-->PNTD-D-25-01115R1

Bridging laboratory and field research: method adjustments to manipulate field-derived *Aedes aegypti*

PLOS Neglected Tropical Diseases

Dear Dr. Gendrin,

Thank you for submitting your manuscript to PLOS Neglected Tropical Diseases. After careful consideration, we feel that it has merit but does not fully meet PLOS Neglected Tropical Diseases's publication criteria as it currently stands. Therefore, we invite you to submit a revised version of the manuscript that addresses the points raised during the review process.

Please submit your revised manuscript within by Apr 17 2026 11:59PM. If you will need more time than this to complete your revisions, please reply to this message or contact the journal office at plosntds@plos.org. When you're ready to submit your revision, log on to https://www.editorialmanager.com/pntd/ and select the 'Submissions Needing Revision' folder to locate your manuscript file.

Please include the following items when submitting your revised manuscript:

* A letter that responds to each point raised by the editor and reviewer(s). You should upload this letter as a separate file labeled 'Response to Reviewers'. This file does not need to include responses to any formatting updates and technical items listed in the 'Journal Requirements' section below.

* A marked-up copy of your manuscript that highlights changes made to the original version. You should upload this as a separate file labeled 'Revised Manuscript with Track Changes'.

* An unmarked version of your revised paper without tracked changes. You should upload this as a separate file labeled 'Manuscript'.

If you would like to make changes to your financial disclosure, competing interests statement, or data availability statement, please make these updates within the submission form at the time of resubmission. Guidelines for resubmitting your figure files are available below the reviewer comments at the end of this letter.

We look forward to receiving your revised manuscript.

Kind regards,

Geoffrey M. Attardo

Academic Editor

PLOS Neglected Tropical Diseases

Paul Mireji

Section Editor

PLOS Neglected Tropical Diseases

Shaden Kamhawi

co-Editor-in-Chief

PLOS Neglected Tropical Diseases

orcid.org/0000-0003-4304-636XX

Paul Brindley

co-Editor-in-Chief

PLOS Neglected Tropical Diseases

orcid.org/0000-0003-1765-0002

**Additional Editor Comments :**

Thank you for submitting your revised manuscript to PLOS Neglected Tropical Diseases. We appreciate the effort you have put into addressing the concerns raised in the previous round of review. Your manuscript has been evaluated by three reviewers, two of whom are satisfied with the revisions and recommend acceptance. However, one reviewer has raised concerns that we believe must be addressed before the manuscript can be accepted for publication. We therefore invite you to submit a major revision.

The reviewer's comments are included in full below, however here are the key points that will require attention:

Isotonic solutions experiment: The reviewer finds the motivation for this experiment to be underdeveloped. The manuscript does not cite relevant literature, does not justify using spermatheca integrity as a readout for tissue integrity more broadly, and does not explain why tissue integrity is relevant to the study's aims. More critically, the conclusion that fertilization status can be assessed post-mortem is not supported by the data, as fertilization itself was never measured. The reviewer recommends removing this experiment from the manuscript. We agree that this is worth serious consideration; if the authors wish to retain it, a substantially stronger justification is required.

Wording error (lines 127–129): The text incorrectly describes the independent variable in the blood feeding experiment as "type of blood meal" when all meals are human-derived, and the variable is, in fact, the method of blood feeding. This should be corrected.

Clarity of experimental design for Figure 1A: It must be made unambiguous whether the Petri dish experiments were performed as part of the same experiment as the Hemotek and lid conditions, or separately. The presentation (either graphically or in the text) should be updated accordingly.

Lines 165–167: The authors should revisit this section, as the statement about strain influencing starvation time is logically inconsistent, given that starvation time is an independent variable. Please clarify the intended meaning.

Figure 2 legend: The legend should be expanded to address the significant difference between strains and to describe the primary findings of panel 2B, so that the figure is self-contained.

When preparing your revision, please provide a point-by-point response letter detailing how each comment has been addressed. Should you disagree with any of the reviewer's suggestions, please provide a clear and evidence-based justification.

We look forward to receiving your revised manuscript.

**********

Note: If the reviewer comments include a recommendation to cite specific previously published works, please review and evaluate these publications to determine whether they are relevant and should be cited. There is no requirement to cite these works unless the editor has indicated otherwise.

**Reviewers' Comments:**

Reviewer's Responses to Questions

**Key Review Criteria Required for Acceptance?**

As you describe the new analyses required for acceptance, please consider the following:

**Methods**

-Are the objectives of the study clearly articulated with a clear testable hypothesis stated?

-Is the study design appropriate to address the stated objectives?

-Is the population clearly described and appropriate for the hypothesis being tested?

-Is the sample size sufficient to ensure adequate power to address the hypothesis being tested?

-Were correct statistical analysis used to support conclusions?

-Are there concerns about ethical or regulatory requirements being met?

Reviewer #1: (No Response)

Reviewer #2: yes

Reviewer #3: See comments below

**********

**Results**

-Does the analysis presented match the analysis plan?

-Are the results clearly and completely presented?

-Are the figures (Tables, Images) of sufficient quality for clarity?

Reviewer #1: (No Response)

Reviewer #2: yes

Reviewer #3: See comments below

**********

**Conclusions**

-Are the conclusions supported by the data presented?

-Are the limitations of analysis clearly described?

-Do the authors discuss how these data can be helpful to advance our understanding of the topic under study?

-Is public health relevance addressed?

Reviewer #1: (No Response)

Reviewer #2: yes

Reviewer #3: See comments below

**********

**Editorial and Data Presentation Modifications?**

Use this section for editorial suggestions as well as relatively minor modifications of existing data that would enhance clarity. If the only modifications needed are minor and/or editorial, you may wish to recommend “Minor Revision” or “Accept”.

Reviewer #1: (No Response)

Reviewer #2: (No Response)

Reviewer #3: (No Response)

**********

**Summary and General Comments**

Use this section to provide overall comments, discuss strengths/weaknesses of the study, novelty, significance, general execution and scholarship. You may also include additional comments for the author, including concerns about dual publication, research ethics, or publication ethics. If requesting major revision, please articulate the new experiments that are needed.

Reviewer #1: This is a marked improvement from the original submission, and I am very pleased to have gotten the chance to see it in its revised form. The authors should be commended for the work that they have done to put this revised version together. I believe it meets the standards for publication as-is. It could, if one were so inclined, benefit from some light copy editing to standardize some of the English (e.g. swapping 'drowned' for 'drown'). However, the text is clear and readable to a native speaker as-is.

Reviewer #2: The authors addressed all the concerns I had

Reviewer #3: I appreciate the improvements the authors have made to the manuscript. I still have some concerns that need to be addressed, however.

There is still insufficient motivation presented for studying the question of whether different isotonic solutions produce different results during spermathecae dissection. The authors state that different isotonic solutions have been used for this purpose, but they do not motivate the study in any way. Is it a relevant question to query whether one solution is better than the other? Is there any literature that suggests that one may be preferable to the other? Is there a reason to think that different solutions could potentially differ in their effects on recently isolated strains? Moreover, the authors indicate that they used spermatheca integrity as a readout for the effects of these solutions on mosquito tissue overall. But what prior research suggests that spermatheca integrity an appropriate readout for overall tissue integrity? Also, perhaps even more important, why is tissue integrity a relevant question in this study? Finally, the authors conclude from these data that female fertilization can be assessed after death given that they dissected spermathecae from drowned females. However, they never actually assessed fertilization, so this statement is not well supported. I strongly suggest the authors simply remove this experiment from the study. It does not appear to offer much benefit and instead introduces substantial confusion.

On line 127-129, the authors indicate that they are testing whether the type of blood meal offered could influence blood feeding success. However, in the next section, they do not evaluate type of blood meal but rather method of blood feeding (since all blood meals in figure 1A are human derived). The wording here should be changed to reflect this.

It is not clear to me whether the Petri dish experiments in figure 1A were performed separately from the other three feeding methods. Based on the way the results are written, the reader is led to believe the Petri dish experiments were performed first and then the Hemotek and lids experiments were performed together in a subsequent experiment. If the Petri dish experiment was performed separately from the other methods, it should be in its own graph in Fig. 1. If all feeding methods presented in Fig. 1A were performed as part of the same experiment, lines 120-143 should be rewritten to make this clear.

In line 165-167, I do not understand what the authors are trying to say. Figure 2A does not appear to present data on blood feeding rate, and how could strain affect blood feeding starvation time when starvation time is an independent variable? The authors need to revisit this sentence and reevaluate what they were trying to say.

In the legend for figure 2, the authors give details as to significant differences and survival rate over time, but do not speak to the significant difference between strain. They also give no detail about the results of 2B. Figure legends should stand alone, therefore, some discussion of the primary findings of figure 2B is important to include in the figure legend.

**********

PLOS authors have the option to publish the peer review history of their article (what does this mean?). If published, this will include your full peer review and any attached files.

If you choose “no”, your identity will remain anonymous but your review may still be made public.

**Do you want your identity to be public for this peer review?** For information about this choice, including consent withdrawal, please see our Privacy Policy.

Reviewer #1: No

Reviewer #2: No

Reviewer #3: No

[NOTE: If reviewer comments were submitted as an attachment file, they will be attached to this email and accessible via the submission site. Please log into your account, locate the manuscript record, and check for the action link "View Attachments". If this link does not appear, there are no attachment files.]

**Figure resubmission:**

-->While revising your submission, we strongly recommend that you use PLOS’s NAAS tool (https://ngplosjournals.pagemajik.ai/artanalysis) to test your figure files. NAAS can convert your figure files to the TIFF file type and meet basic requirements (such as print size, resolution), or provide you with a report on issues that do not meet our requirements and that NAAS cannot fix.--> -->

After uploading your figures to PLOS’s NAAS tool - https://ngplosjournals.pagemajik.ai/artanalysis, NAAS will process the files provided and display the results in the "Uploaded Files" section of the page as the processing is complete. If the uploaded figures meet our requirements (or NAAS is able to fix the files to meet our requirements), the figure will be marked as "fixed" above. If NAAS is unable to fix the files, a red "failed" label will appear above. When NAAS has confirmed that the figure files meet our requirements, please download the file via the download option, and include these NAAS processed figure files when submitting your revised manuscript.-->

**Reproducibility:**

To enhance the reproducibility of your results, we recommend that authors of applicable studies deposit laboratory protocols in protocols.io, where a protocol can be assigned its own identifier (DOI) such that it can be cited independently in the future. Additionally, PLOS ONE offers an option to publish peer-reviewed clinical study protocols. Read more information on sharing protocols at https://plos.org/protocols?utm_medium=editorial-email&utm_source=authorletters&utm_campaign=protocols

-->

# Author comment: Author response to Decision Letter 2

**DOI:** 10.1371/journal.pntd.0013339.r004

24 Mar 2026

Attachment Submitted filename: Response_to_reviewers_auresp_2.docx

# Peer review: Decision Letter 2

**DOI:** 10.1371/journal.pntd.0013339.r005

**Authors:** Paul Mireji

4 May 2026

Dear Dr Gendrin,

We are pleased to inform you that your manuscript 'Bridging laboratory and field research: method adjustments to blood feed field-derived *Aedes aegypti*' has been provisionally accepted for publication in PLOS Neglected Tropical Diseases.

Before your manuscript can be formally accepted you will need to complete some formatting changes, which you will receive in a follow up email. A member of our team will be in touch with a set of requests.

Please note that your manuscript will not be scheduled for publication until you have made the required changes, so a swift response is appreciated.

IMPORTANT: The editorial review process is now complete. PLOS will only permit corrections to spelling, formatting or significant scientific errors from this point onwards. Requests for major changes, or any which affect the scientific understanding of your work, will cause delays to the publication date of your manuscript.

Should you, your institution's press office or the journal office choose to press release your paper, you will automatically be opted out of early publication. We ask that you notify us now if you or your institution is planning to press release the article. All press must be co-ordinated with PLOS.

Thank you again for supporting Open Access publishing; we are looking forward to publishing your work in PLOS Neglected Tropical Diseases.

Best regards,

Geoffrey M. Attardo

Academic Editor

PLOS Neglected Tropical Diseases

Paul Mireji

Section Editor

PLOS Neglected Tropical Diseases

Shaden Kamhawi

co-Editor-in-Chief

PLOS Neglected Tropical Diseases

orcid.org/0000-0003-4304-636XX

Paul Brindley

co-Editor-in-Chief

PLOS Neglected Tropical Diseases

orcid.org/0000-0003-1765-0002

***********************************************************

Thank you for the resubmission of your article and for addressing the comments provided by the reviewer. We are happy to let know you know that the manuscript has been accepted for publication.

p.p1 {margin: 0.0px 0.0px 0.0px 0.0px; line-height: 16.0px; font: 14.0px Arial; color: #323333; -webkit-text-stroke: #323333}span.s1 {font-kerning: none

Reviewer's Responses to Questions

**Key Review Criteria Required for Acceptance?**

As you describe the new analyses required for acceptance, please consider the following:

**Methods**

-Are the objectives of the study clearly articulated with a clear testable hypothesis stated?

-Is the study design appropriate to address the stated objectives?

-Is the population clearly described and appropriate for the hypothesis being tested?

-Is the sample size sufficient to ensure adequate power to address the hypothesis being tested?

-Were correct statistical analysis used to support conclusions?

-Are there concerns about ethical or regulatory requirements being met?

Reviewer #3: All issues addressed

**********

**Results**

-Does the analysis presented match the analysis plan?

-Are the results clearly and completely presented?

-Are the figures (Tables, Images) of sufficient quality for clarity?

Reviewer #3: All issues addressed

**********

**Conclusions**

-Are the conclusions supported by the data presented?

-Are the limitations of analysis clearly described?

-Do the authors discuss how these data can be helpful to advance our understanding of the topic under study?

-Is public health relevance addressed?

Reviewer #3: All issues addressed

**********

**Editorial and Data Presentation Modifications?**

Use this section for editorial suggestions as well as relatively minor modifications of existing data that would enhance clarity. If the only modifications needed are minor and/or editorial, you may wish to recommend “Minor Revision” or “Accept”.

Reviewer #3: line 67: have enabled determination of...

line 176: post-hoc analyses on starvation...

**********

**Summary and General Comments**

Use this section to provide overall comments, discuss strengths/weaknesses of the study, novelty, significance, general execution and scholarship. You may also include additional comments for the author, including concerns about dual publication, research ethics, or publication ethics. If requesting major revision, please articulate the new experiments that are needed.

Reviewer #3: All issues addressed

**********

PLOS authors have the option to publish the peer review history of their article (what does this mean?). If published, this will include your full peer review and any attached files.

If you choose “no”, your identity will remain anonymous but your review may still be made public.

**Do you want your identity to be public for this peer review?** For information about this choice, including consent withdrawal, please see our Privacy Policy.

Reviewer #3: No

# Editor report: Acceptance letter

**DOI:** 10.1371/journal.pntd.0013339.r006

**Authors:** Paul Mireji

Dear Dr Gendrin,

We are delighted to inform you that your manuscript, "Bridging laboratory and field research: method adjustments to blood feed field-derived *Aedes aegypti*," has been formally accepted for publication in PLOS Neglected Tropical Diseases.

We have now passed your article onto the PLOS Production Department who will complete the rest of the publication process. All authors will receive a confirmation email upon publication.

The corresponding author will soon be receiving a typeset proof for review, to ensure errors have not been introduced during production. Please review the PDF proof of your manuscript carefully, as this is the last chance to correct any scientific or type-setting errors. Please note that major changes, or those which affect the scientific understanding of the work, will likely cause delays to the publication date of your manuscript. Note: Proofs for Front Matter articles (Editorial, Viewpoint, Symposium, Review, etc...) are generated on a different schedule and may not be made available as quickly.

Soon after your final files are uploaded, the early version of your manuscript will be published online unless you opted out of this process. The date of the early version will be your article's publication date. The final article will be published to the same URL, and all versions of the paper will be accessible to readers.

For Research Articles, you will receive an invoice from PLOS for your publication fee after your manuscript has reached the completed accept phase. If you receive an email requesting payment before acceptance or for any other service, this may be a phishing scheme. Learn how to identify phishing emails and protect your accounts at https://explore.plos.org/phishing.

Thank you again for supporting open-access publishing; we are looking forward to publishing your work in PLOS Neglected Tropical Diseases.

Best regards,

Shaden Kamhawi

co-Editor-in-Chief

PLOS Neglected Tropical Diseases

Paul Brindley

co-Editor-in-Chief

PLOS Neglected Tropical Diseases
